# Assessment of glycemic susceptibility across multiple urological and reproductive disorders

**DOI:** 10.1186/s13098-024-01404-x

**Published:** 2024-07-15

**Authors:** Xiongfeng Zeng, Zhen Li, Lede Lin, Xin Wei

**Affiliations:** 1 Department of Urology, Institute of Urology, West China Hospital, Sichuan University, Chengdu, Sichuan China; 2 Department of Urology, Hunan Provincial People’s Hospital, The First Affiliated Hospital of Hunan Normal University, Changsha, Hunan China

**Keywords:** Glycemic traits, Urological/reproductive diseases, Type II diabetes

## Abstract

**Objective:**

To test the glycemic susceptibility in three urological cancers and eight urological/reproductive diseases using the Mendelian randomization (MR) method.

**Materials and methods:**

Two-sample MR was applied to investigate the causal role of three glycemic traits (type II diabetes, fasting glucose and glycated hemoglobin (HbA1c)) in eleven urological/reproductive diseases (kidney cancer, bladder cancer, prostate cancer, kidney/ureter stone, urinary incontinence, benign prostatic hyperplasia, erectile dysfunction, female infertility, male infertility, abnormal spermatozoa and polycystic ovary syndrome). Further multivariate MR (MVMR) and mediating analysis were performed to investigate the associations.

**Results:**

Among all the 11 diseases, type II diabetes was positively associated with erectile dysfunction, which was stable across both cohorts [odds ratio (OR): 1.59, 95% confidence interval (CI): 1.15–2.20, *P* = 0.005 for FinnGen Biobank and OR: 1.14, 95% CI: 1.08–1.21, *P* < 0.001 for the other cohort]. Also, type II diabetes was negatively associated with male infertility (OR: 0.57, 95% CI: 0.39–0.84, *P* = 0.005). In addition, all three glycemic traits were observed to be positively associated with polycystic ovary syndrome (OR: 2.36, 95% CI: 1.16–4.76, *P* = 0.017 for fasting glucose per mmol/L; OR: 3.04, 95% CI: 1.10–8.39, *P* = 0.032 for HbA1c per percentage; and OR: 1.21, 95% CI: 1.00-1.46, *P* = 0.046 for type II diabetes). Mediating analysis confirmed the effect of type II diabetes on these diseases.

**Conclusions:**

There existed glycemic susceptibility in erectile dysfunction, male infertility and polycystic ovary syndrome. We could not conclude stable glycemic susceptibility in other urological/reproductive diseases.

**Supplementary Information:**

The online version contains supplementary material available at 10.1186/s13098-024-01404-x.

## Introduction

Diabetes mellitus (DM) is a metabolic disease, which has been considered one of the most prevalent chronic diseases impacting all around the world [1, 2]. Among all DM individuals, type II diabetes accounts for over 90% [1], which was thought to influence numerous other chronic diseases, including the occurrence of kidney cancer [3], bladder cancer [3–5], erectile dysfunction [6], benign prostatic hyperplasia (BPH) [7, 8], bladder dysfunction [9], colorectal cancer [10], sexual dysfunction [11] and so on. However, all these associations were based on observational studies, and conducting randomized controlled trials (RCTs) to explore the causal relationship between type II diabetes and other diseases is impractical. Because it was unethical to randomize participants to suffer from DM or not, and the period to observe enough endpoint events like bladder cancer was unclear. Hence, researches based on genetic epidemiology design are urgently needed because they solve the problem of causal uncertainty [12–16].

To overcome the shortcomings of conventional epidemiological studies, we performed such a study with Mendelian randomization (MR) design to test the glycemic susceptibility in three urological cancers and eight urological/reproductive diseases.

## Materials and methods

This MR study utilized data from previous genome-wide association studies (GWAS) with large participants. All the data were manually curated by the MRC Integrative Epidemiology Unit (IEU) at the University of Bristol, which could be accessed through the OpenGWAS database API [17, 18]. Only the European population was included. The study complied with the Declaration of Helsinki. As the data in our study were publicly available, informed consents and institutional review board approval were exempted.

### Glycemic traits

We retrieved summary statistics of type II diabetes, fasting glucose and glycated hemoglobin (HbA1c) from recent GWAS, with high quality and large sample size [19, 20], Table S1). Although fasting glucose and HbA1c traits were from participants free of diabetes, they correlated well with type II diabetes [19, 20], Table S2). The total sample size of fasting glucose, HbA1c and type II diabetes was 200,622, 146,806 and 655,666 respectively (Table S1). Only significant single nucleotide polymorphisms (SNPs) with P value < 5e-08 and without linkage disequilibrium (r^2 = 0.001 and kb = 10,000) were included in our analysis. Ultimately we just extracted 66, 74 and 118 significant SNPs for fasting glucose, HbA1c and type II diabetes. Further details about the three glycemic traits were provided in Table S3-S5.

### Eleven urological traits

In this study, we enrolled eleven urological/reproductive diseases from recent GWAS, comprising kidney cancer, bladder cancer, prostate cancer, kidney/ureter stone, urinary incontinence, BPH, erectile dysfunction, female infertility, male infertility, abnormal spermatozoa and polycystic ovary syndrome (Table S1). For several diseases, GWAS derived from different consortia focused on the same disease. We included all of them in our analysis. The sources were mainly from the UK Biobank, FinnGen Biobank and some other consortia (Table S1).

### Statistical analysis

All the MR analysis was completed on R software (4.1.2). TwoSampleMR was the main package. When extracting significant SNPs (*P* < 5e-08 and without linkage disequilibrium: r^2 = 0.001 and kb = 10,000), we calculated the F-statistic based on the beta and standard error of each SNP, according to a previous publication [21]. F-statistic > 10 was considered an instrument variable with sufficient strength. Five MR methods were used for the combination of all SNPs: inverse variance weighted (IVW), IVW (multiplicative random effects), MR Egger, MR Egger (bootstrap) and weighted median. Results were reported as beta or odds ratio (OR) with 95% confidence interval (95% CI). Heterogeneity and pleiotropy tests were also conducted. If significant heterogeneity was detected, the IVW (multiplicative random effects) method was recommended. If significant pleiotropy was detected, the Mendelian Randomization Pleiotropy RESidual Sum and Outlier (MR-PRESSO) and the MR Egger method were recommended. The p-value threshold was set to 0.05 on two sides.

**Fig. 1:**
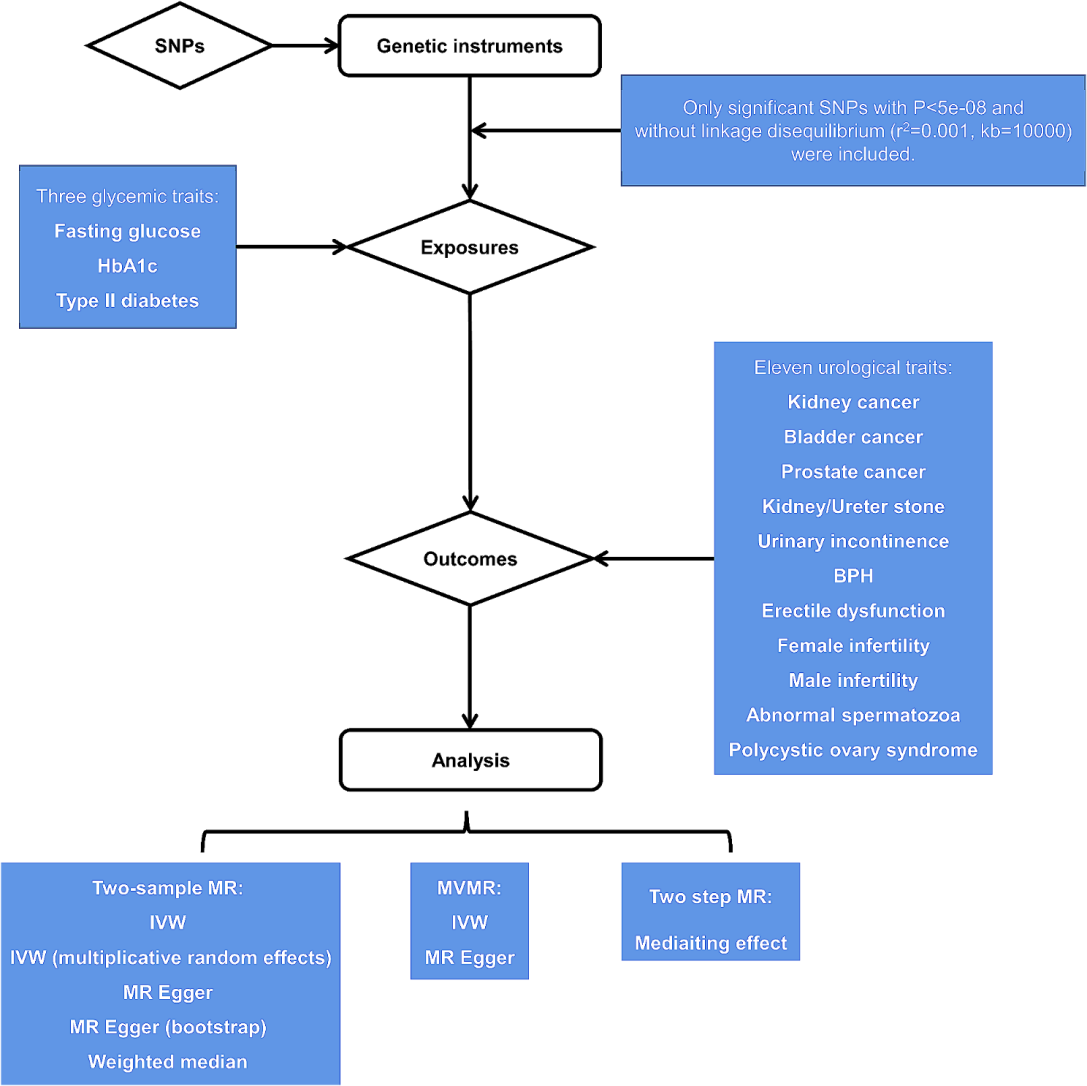
Study flow chart to test the causal associations between three glycemic traits and eleven urological traits SNP: single nucleotide polymorphisms; HbA1c: glycated hemoglobin; BPH: benign prostatic hyperplasia; MR: Mendelian randomization; MVMR: multivariate Mendelian randomization; IVW: inverse variance weighted; MR Egger: Egger’s regression for Mendelian randomization

To go a step further, multivariate MR (MVMR) was performed to identify the effect of exposure-specific SNPs on outcomes. Two main MVMR methods were utilized: IVW and MR Egger. After conducting two-sample MR and MVMR, a two-step MR analysis was performed to investigate the mediating effect of type II diabetes on urological disease outcomes. The mediating effect was defined as the product of two-sample MR results between exposure and mediator and between mediator and outcome. The general study flow chart was depicted in Fig. 1.

## Results

As shown in Fig. 1, the study mainly contained three parts:

1. causal associations between three glycemic traits and eleven urological traits based on two-sample MR analysis;
2. MVMR analysis to explore the adjusted effect of three glycemic traits from significant outcomes in part (1);
3. mediating analysis to describe the mediating effect of type II diabetes on urological outcomes from the results of part (1) and part (2).

### Causal associations between three glycemic traits and eleven urological/reproductive traits

We included 11 urological/reproductive diseases: kidney cancer, bladder cancer, prostate cancer, kidney/ureter stone, urinary incontinence, BPH, erectile dysfunction, female infertility, male infertility, abnormal spermatozoa and polycystic ovary syndrome (Table S1, Table S6-S16). Several diseases were derived from different GWAS, so we involved all of them in our two-sample MR analysis to test the stability of our results.

**Fig. 2:**
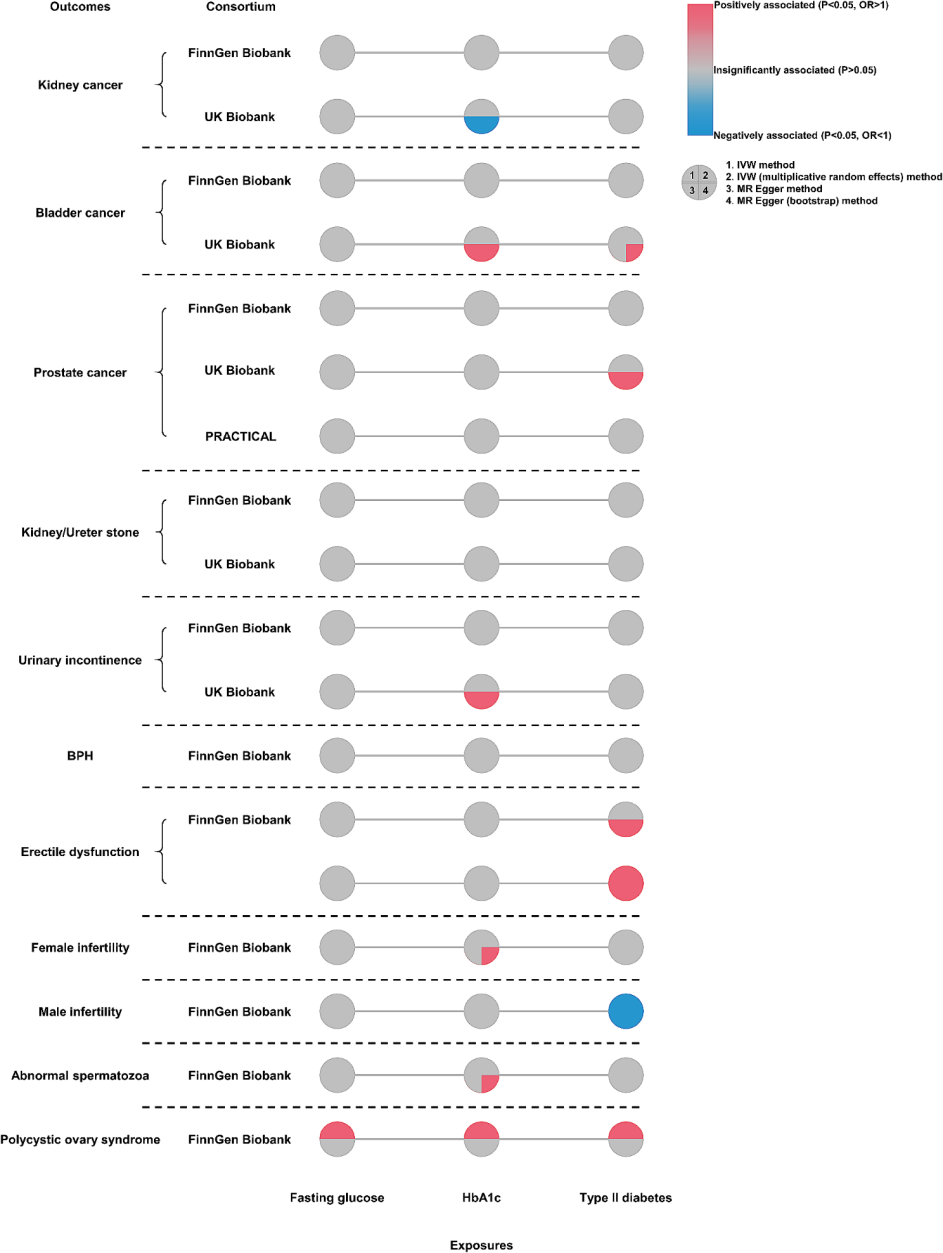
Causal associations between three exposures and eleven urological outcomes based on two-sample MR. MR: Mendelian randomization; HbA1c: glycated hemoglobin; BPH: benign prostatic hyperplasia; PRACTICAL: Prostate Cancer Association Group to Investigate Cancer Associated Alterations in the Genome; IVW: inverse variance weighted; MR Egger: Egger’s regression for Mendelian randomization; OR: odds ratio

We discovered a certain glycemic trait was associated with several diseases in part of GWAS, but the results were not so stable across all the consortia. For example, type II diabetes was positively associated with prostate cancer just in the cohort of UK Biobank in the method of MR Egger (OR: 1.01, 95% CI: 1.00-1.01, *P* = 0.021, Fig. 2 and Table S8). Type II diabetes was negatively associated with male infertility (FinnGen Biobank) across all the MR methods (Fig. 2 and Table S14).

Generally, glycemic susceptibility might exist in kidney cancer (negatively impacted by HbA1c, Fig. 2 and Table S6), bladder cancer (positively impacted by HbA1c, Fig. 2 and Table S7), prostate cancer (positively impacted by type II diabetes, Fig. 2 and Table S8), erectile dysfunction (positively impacted by type II diabetes, Fig. 2 and Table S12), male infertility (negatively impacted by type II diabetes, Fig. 2 and Table S14) and polycystic ovary syndrome (positively impacted by fasting glucose, HbA1c and type II diabetes, Fig. 2 and Table S16).

Among all the analysis in Fig. 2, we found type II diabetes was positively associated with erectile dysfunction across all cohorts. As the heterogeneity and pleiotropy test shown in Table S12, the MR Egger method was prioritized for erectile dysfunction (FinnGen Biobank) (OR: 1.59; 95% CI: 1.15–2.20; *P* = 0.005, Table S12) and IVW method for erectile dysfunction (OR: 1.14; 95% CI: 1.08–1.21; *P* < 0.001, Table S12). While type II diabetes was negatively associated with male infertility (OR: 0.57; 95% CI: 0.39–0.84; *P* = 0.005 for MR Egger method, Table S14). We also discovered that polycystic ovary syndrome was positively impacted by all three glycemic traits in the IVW method (Fig. 2 and Table S16). The results were stable and the glycemic susceptibility in those urological diseases could be established.

### The direct effect of three glycemic traits on eleven urological/reproductive traits based on MVMR

Based on the two-sample MR results, we performed MVMR analysis for those cohorts with significant two-sample MR results (Figure S1-S10). We again found type II diabetes was still positively associated with erectile dysfunction (Figure S6) and negatively associated with male infertility (Figure S8) in both IVW and MR Egger methods.

### Mediating analysis to investigate the effect of type II diabetes on urological/reproductive outcomes

We conducted a two-step MR analysis to assess the mediating effects of type II diabetes on urological/reproductive outcomes based on previous results. Mediating analysis showed that type II diabetes mediated the effect of fasting glucose and HbA1c on four urological/reproductive diseases: prostate cancer, erectile dysfunction, male infertility and polycystic ovary syndrome (Table 1).

**Table 1:** Two step MR to assess the mediator effects of type II diabetes on urological outcomes

Effects	Exposures	Mediators	Outcomes	SNPs	OR	95% CI	*P* value
Mediating^1^	Fasting glucose	Type II diabetes		55	5.65	2.99–10.68	< 0.001
Mediating^2^	HbA1c	Type II diabetes		56	2.32	1.65–3.26	< 0.001
**Mediator effect of type II diabetes on prostate cancer (UK Biobank).**							
Mediating^3^		Type II diabetes	Prostate cancer	113	1.01	1.00−1.01	0.021
**Mediator effect of type II diabetes on erectile dysfunction (FinnGen Biobank).**							
Mediating^3^		Type II diabetes	Erectile dysfunction	114	1.59	1.15–2.20	0.005
**Mediator effect of type II diabetes on erectile dysfunction.**							
Mediating^4^		Type II diabetes	Erectile dysfunction	115	1.14	1.08–1.21	< 0.001
**Mediator effect of type II diabetes on male infertility (FinnGen Biobank).**							
Mediating^3^		Type II diabetes	Male infertility	114	0.57	0.39–0.84	0.005
**Mediator effect of type II diabetes on polycystic ovary syndrome (FinnGen Biobank).**							
Mediating^1^		Type II diabetes	Polycystic ovary syndrome	114	1.21	1.00−1.46	0.046

1: Based on IVW (multiplicative random effects) method due to significant heterogeneity

2: Based on MR-PRESSO method due to significant pleiotropy and detectable outliers

3: Based on MR Egger method due to significant pleiotropy

4: Based on IVW method

MR: Mendelian randomization; HbA1c: glycated hemoglobin; SNP: single nucleotide polymorphisms; IVW: inverse variance weighted; MR-PRESSO: Mendelian Randomization Pleiotropy RESidual Sum and Outlier; MR Egger: Egger’s regression for Mendelian randomization

## Discussion

**Fig. 3:**
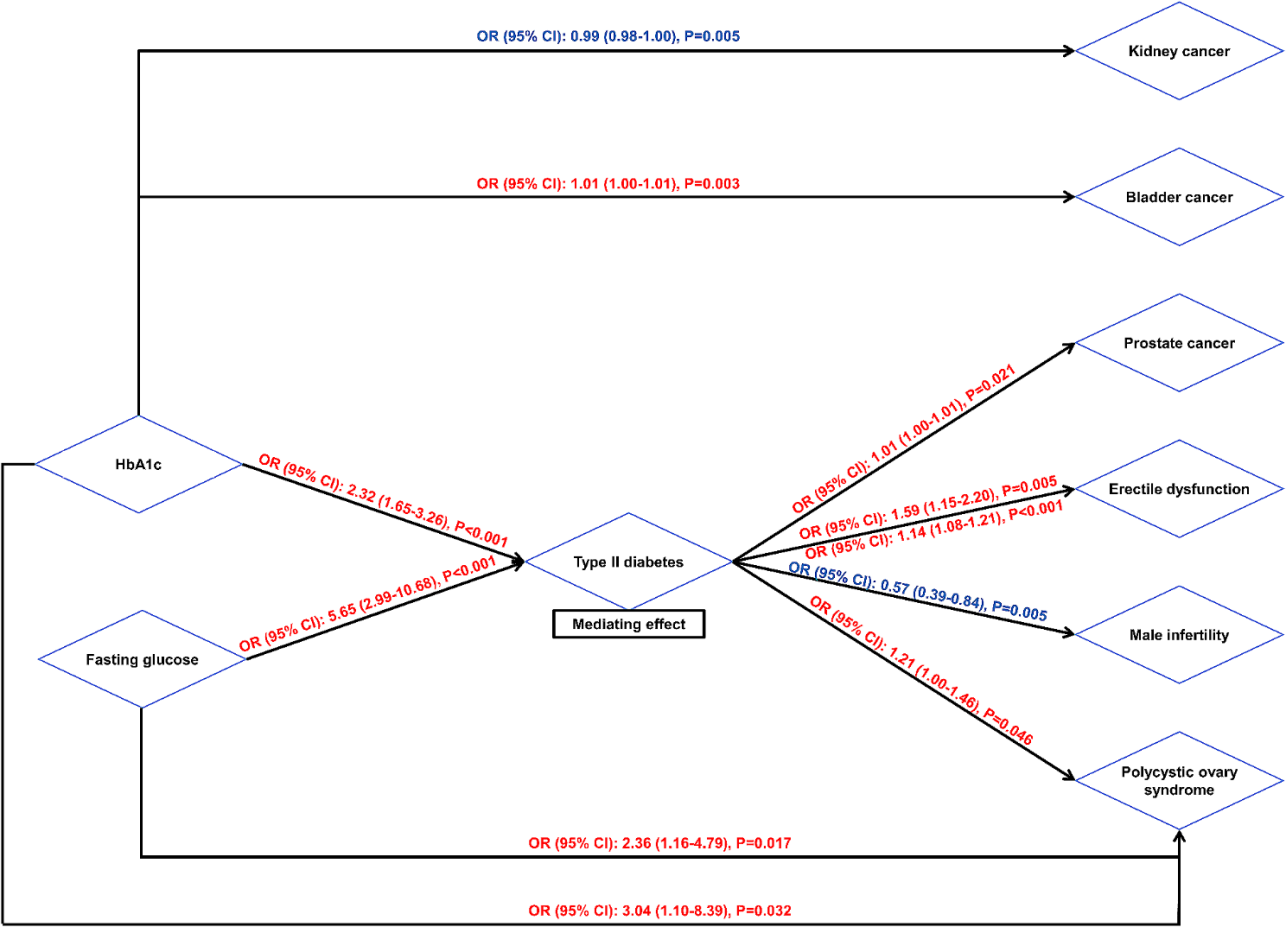
The mediating effects of type II diabetes on urological outcomes

In this study, we explored the glycemic susceptibility in eleven urological/reproductive diseases, including kidney cancer, bladder cancer, prostate cancer, kidney/ureter stone, urinary incontinence, BPH, erectile dysfunction, male infertility, female infertility, abnormal spermatozoa and polycystic ovary syndrome. We discovered that several diseases might have glycemic susceptibility like kidney cancer (negative association, Fig. 2), bladder cancer (positive association, Fig. 2), and prostate cancer (positive association, Fig. 2). However, those associations were not so stable and were only observed in partial cohorts. Moreover, the OR value was unexpectedly low (approximately equal to “1”, Fig. 3) in spite of a significant P value. We did find type II diabetes was positively associated with erectile dysfunction, which was stable across both cohorts [OR: 1.59, 95% CI: 1.15–2.20, *P* = 0.005 for erectile dysfunction (FinnGen Biobank) and OR: 1.14, 95% CI: 1.08–1.21, *P* < 0.001 for erectile dysfunction, Fig. 3]. Also, type II diabetes was negatively associated with male infertility (OR: 0.57, 95% CI: 0.39–0.84, *P* = 0.005, Fig. 3). In addition, all three glycemic traits were observed to be positively associated with polycystic ovary syndrome (OR: 2.36, 95% CI: 1.16–4.76, *P* = 0.017 for fasting glucose per mmol/L; OR: 3.04, 95% CI: 1.10–8.39, *P* = 0.032 for HbA1c per percentage; and OR: 1.21, 95% CI: 1.00-1.46, *P* = 0.046 for type II diabetes, Fig. 3). We depicted these associations in Fig. 3, which was meaningful for future researches.

The relationship between diabetes and cancer has been debated over the years [22, 23]. However, nearly all studies were based on cohort study design, which could not totally avoid confounder bias. It is now generally considered diabetes promotes tumorigenesis in a majority of organs, including the stomach, pancreas, liver, kidney, bladder, breast, endometrium, colorectal, head and neck [3, 10, 22, 23]. However, an inverse association between diabetes and prostate cancer was observed [22, 23]. We conducted such an MR study to prove the causal associations between glycemic traits and three urological cancers. Although several significant associations were observed, the results were not so stable that validated glycemic susceptibility could not be established in the three urological cancers. An unexpected positive relationship was found between type II diabetes and prostate cancer, although the results were not so robust and the actual causality should be doubted. Despite conventional epidemiology studies confirming the increased risk of kidney cancer in different diabetic populations [3, 24–27], we did not find such shreds of evidence from the point of genetic epidemiology, and even an unstable negative relationship was discovered. Hence, the association should be interpreted cautiously.

Recently, a renowned clinical research has been published to illustrate the role of one type of anti-diabetic medication, SGLT2 inhibitors, in tumorigenesis of bladder cancer [5]. The study concluded that the medication did not increase the risk of bladder cancer. We might need to think about that in another way. Since several anti-diabetic medications reported the occurrence of bladder cancer [5, 22, 28], we should ponder on the relevance between glycemic metabolism and bladder cancer. Maybe the influence of glycemic metabolism could induce bladder tumorigenesis and there might exist a genetic association between the two entities. Recently, a large cohort study from UK Biobank implied diabetes and higher HbA1c levels were associated with an increased risk of bladder cancer [23]. However, in our study, we could not find a robust association between glycemic traits and bladder cancer. Only a weak causal association was found in the UK Biobank cohort (Fig. 2 and Table S7). The phenomena reminded us that there might exist potential confounders impacting glycemic metabolism and bladder cancer. We were not sure that a causal association could be confirmed.

Type II diabetes has been thought of as a risk factor in numerous urological/reproductive benign diseases, including kidney stones [29], bladder dysfunction (9), BPH [7, 8] and infertility-related diseases [6, 11, 30–34]. Nevertheless, they were generally based on clinical cohort studies or conventional epidemiological population studies, which might be obscured by confounders. In our study, we could only conclude that glycemic traits influenced erectile dysfunction (OR: 1.59, 95% CI: 1.15–2.20, *P* = 0.005 for type II diabetes in FinnGen Biobank cohort; OR: 1.14, 95% CI: 1.08–1.21, *P* < 0.001 for type II diabetes in the other cohort, Fig. 3 and Table S12), male infertility (OR: 0.57, 95% CI: 0.39–0.84, *P* = 0.005 for type II diabetes, Fig. 3 and Table S14) and polycystic ovary syndrome (OR: 2.36, 95% CI: 1.16–4.76, *P* = 0.017 for fasting glucose per mmol/L; OR: 3.04, 95% CI: 1.10–8.39, *P* = 0.032 for HbA1c per percentage; and OR: 1.21, 95% CI: 1.00-1.46, *P* = 0.046 for type II diabetes, Fig. 3 and Table S16). MVMR results indicated that type II diabetes was able to negatively impact polycystic ovary syndrome in a direct way (Figure S8), but type II diabetes could not impact erectile dysfunction in both cohorts (Figure S5 and Figure S6). Mediating analysis revealed that type II diabetes mediated the effect of fasting glucose and HbA1c on erectile dysfunction, male infertility and polycystic ovary syndrome (Table 1; Fig. 3). These were novel findings from the genetic level and further studies might focus on the glycemic susceptibility of those diseases. Maybe studies on the reproduction of non-mammalian animal models could provide certain clues [35]. On the other hand, we observed different effects of type II diabetes on male and female infertility (Fig. 2). The phenomenon indicated the impact of gender differences in the glycemic susceptibility of infertility.

The study for the first time stressed the glycemic susceptibility in erectile dysfunction, male infertility and polycystic ovary syndrome among all the eleven urological/reproductive diseases. However, we should admit several shortcomings. We only included the European population in our study, so the application would be restricted. Also, further researches should focus on the mechanism of glycemic susceptibility of those diseases.

To summarize, there existed glycemic susceptibility in erectile dysfunction, male infertility and polycystic ovary syndrome. We could not conclude stable glycemic susceptibility in other urological/reproductive diseases.

HbA1c: glycated hemoglobin; OR: odds ratio; 95% CI: 95% confidence interval.

### Electronic supplementary material

Below is the link to the electronic supplementary material.

Supplementary Material 1

Supplementary Material 2

## Data Availability

All data generated or analyzed during this study are included in this published article and its supplementary information files.
