# Supplementary material for: Assessment of glycemic susceptibility across multiple urological and reproductive disorders
Source: Diabetol Metab Syndr. 2024 Jul 15;16:162. doi: 10.1186/s13098-024-01404-x (PMC11247903; doi:10.1186/s13098-024-01404-x)
Supplement: Supplementary file 1 — Supplementary Material 1 [file 13098_2024_1404_MOESM1_ESM.docx]

**Table S1. Details about included traits from previous GWAS**

| **Phenotypes** | **Traits** | **Sex** | **Population** | **Sample size** | **Units** | **SNPs** | **Consortium** | **ID*** | **PMID** | **Year** |
| --- | --- | --- | --- | --- | --- | --- | --- | --- | --- | --- |
| Exposures | Fasting glucose | Males and females | European | 200,622 | mmol/L | 31,008,728 | MAGIC | ebi-a-GCST90002232 | 34059833 | 2021 |
|  | HbA1c | Males and females | European | 146,806 | % | 30,649,064 | MAGIC | ebi-a-GCST90002244 | 34059833 | 2021 |
|  | Type II diabetes | Males and females | European | Case: 61,714  Control: 593,952 | Binary | 5,030,727 | eQTLGen | ebi-a-GCST006867 | 30054458 | 2018 |
| Outcomes | Kidney cancer | Males and females | European | Case: 971  Control: 217,821 | Binary | 16,380,466 | FinnGen Biobank | [finn-b-C3_KIDNEY_](https://gwas.mrcieu.ac.uk/datasets/finn-b-C3_KIDNEY_NOTRENALPELVIS/)  [NOTRENALPELVIS](https://gwas.mrcieu.ac.uk/datasets/finn-b-C3_KIDNEY_NOTRENALPELVIS/) | - | 2021 |
|  |  | Males and females | European | Case: 1,114  Control: 461,896 | Binary | 9,851,867 | UK Biobank | [ukb-b-1316](https://gwas.mrcieu.ac.uk/datasets/ukb-b-1316/) | - | 2018 |
|  | Bladder cancer | Males and females | European | Case: 1,279  Control: 372,016 | Binary | 9,904,926 | UK Biobank | [ieu-b-4874](https://gwas.mrcieu.ac.uk/datasets/ieu-b-4874/) | - | 2021 |
|  |  | Males and females | European | Case: 1,115  Control: 217,677 | Binary | 16,380,466 | FinnGen Biobank | [finn-b-C3_](https://gwas.mrcieu.ac.uk/datasets/finn-b-C3_BLADDER/)  [BLADDER](https://gwas.mrcieu.ac.uk/datasets/finn-b-C3_BLADDER/) | - | 2021 |
|  | Prostate cancer | Males | European | Case: 9,132  Control: 173,493 | Binary | - | UK Biobank | [ieu-b-4809](https://gwas.mrcieu.ac.uk/datasets/ieu-b-4809/) | - | 2021 |
|  |  | Males | European | Case: 79,148  Control: 61,106 | Binary | 20,346,368 | PRACTICAL | ieu-b-85 | 29892016 | 2018 |
|  |  | Males | European | Case: 6,311  Control: 88,902 | Binary | 16,378,835 | FinnGen Biobank | [finn-b-C3_](https://gwas.mrcieu.ac.uk/datasets/finn-b-C3_PROSTATE/)  [PROSTATE](https://gwas.mrcieu.ac.uk/datasets/finn-b-C3_PROSTATE/) | - | 2021 |
|  | Kidney/Ureter stone | Males and females | European | Case: 4,969  Control: 213,445 | Binary | 16,380,464 | FinnGen Biobank | [finn-b-N14_](https://gwas.mrcieu.ac.uk/datasets/finn-b-N14_CALCUKIDUR/)  [CALCUKIDUR](https://gwas.mrcieu.ac.uk/datasets/finn-b-N14_CALCUKIDUR/) | - | 2021 |
|  |  | Males and females | European | Case: 3,625  Control: 459,308 | Binary | 9,851,867 | UK Biobank | ukb-b-8297 | - | 2018 |
|  | Urinary incontinence | Males and females | European | Case: 1,357  Control: 202,910 | Binary | 16,380,434 | FinnGen Biobank | [finn-b-R18_UNSPE_](https://gwas.mrcieu.ac.uk/datasets/finn-b-R18_UNSPE_URINARY_INCONTINENCE/)  [URINARY_](https://gwas.mrcieu.ac.uk/datasets/finn-b-R18_UNSPE_URINARY_INCONTINENCE/)  [INCONTINENCE](https://gwas.mrcieu.ac.uk/datasets/finn-b-R18_UNSPE_URINARY_INCONTINENCE/) | - | 2021 |
|  |  | Males and females | European | Case: 1,624  Control: 461,309 | Binary | 9,851,867 | UK Biobank | [ukb-b-8517](https://gwas.mrcieu.ac.uk/datasets/ukb-b-8517/) | - | 2018 |
|  | BPH | Males | European | Case: 13,118  Control: 72,799 | Binary | 16,378,414 | FinnGen Biobank | [finn-b-N14_](https://gwas.mrcieu.ac.uk/datasets/finn-b-N14_PROSTHYPERPLA/)  [PROSTHYPERPLA](https://gwas.mrcieu.ac.uk/datasets/finn-b-N14_PROSTHYPERPLA/) | - | 2021 |
|  | Erectile dysfunction | Males | European | Case: 1,154  Control: 94,024 | Binary | 16,378,833 | FinnGen Biobank | [finn-b-ERECTILE_](https://gwas.mrcieu.ac.uk/datasets/finn-b-ERECTILE_DYSFUNCTION/)  [DYSFUNCTION](https://gwas.mrcieu.ac.uk/datasets/finn-b-ERECTILE_DYSFUNCTION/) | - | 2021 |
|  |  | Males | European | Case: 6,175  Control: 217,630 | Binary | 9,310,196 | - | [ebi-a-GCST006956](https://gwas.mrcieu.ac.uk/datasets/ebi-a-GCST006956/) | 30583798 | 2019 |
|  | Female infertility | Females | European | Case: 6,481  Control: 68,969 | Binary | 16,377,038 | FinnGen Biobank | [finn-b-N14_](https://gwas.mrcieu.ac.uk/datasets/finn-b-N14_FEMALEINFERT/)  [FEMALEINFERT](https://gwas.mrcieu.ac.uk/datasets/finn-b-N14_FEMALEINFERT/) | - | 2021 |
|  | Male infertility | Males | European | Case: 680  Control: 72,799 | Binary | 16,377,329 | FinnGen Biobank | [finn-b-N14_](https://gwas.mrcieu.ac.uk/datasets/finn-b-N14_MALEINFERT/)  [MALEINFERT](https://gwas.mrcieu.ac.uk/datasets/finn-b-N14_MALEINFERT/) | - | 2021 |
|  | Abnormal spermatozoa | Males | European | Case: 915  Control: 209,006 | Binary | 16,380,442 | FinnGen Biobank | finn-b-R18_  ABNORMAL_  SPERMATOZ | - | 2021 |
|  | Polycystic ovary syndrome | Females | European | Case: 642  Control: 118,228 | Binary | 16,379,676 | FinnGen Biobank | [finn-b-E4_POCS](https://gwas.mrcieu.ac.uk/datasets/finn-b-E4_POCS/) | - | 2021 |

GWAS: genome-wide association study; SNP: single nucleotide polymorphisms; HbA1c: glycated hemoglobin; BPH: benign prostatic hyperplasia; MAGIC: Meta-Analyses of Glucose and Insulin-Related Traits Consortium; PRACTICAL: Prostate Cancer Association Group to Investigate Cancer Associated Alterations in the Genome.

*: All the datasets could be accessed through the corresponding ID number from the OpenGWAS database (https://gwas.mrcieu.ac.uk).

**Table S2. Associations between fasting glucose, HbA1c and type II diabetes utilizing two-sample MR.**

| **Exposures** | **Outcomes** | **SNPs** | **MR methods** | **Beta** | **Standard error** | **P value** | **Q_heterogeneity_** | **P_heterogeneity_** | **P_pleiotropy_** |
| --- | --- | --- | --- | --- | --- | --- | --- | --- | --- |
| Type II diabetes | Fasting glucose | 115 | IVW | **0.1174** | **0.0131** | **<0.001** | **3124.91** | **<0.001** | 0.979 |
|  |  |  | IVW (multiplicative random effects) | **0.1174** | **0.0131** | **<0.001** |  |  |  |
|  |  |  | MR Egger | **0.1182** | **0.0301** | **<0.001** | **3124.89** | **<0.001** |  |
|  |  |  | MR Egger (bootstrap) | **0.1158** | **0.0092** | **<0.001** |  |  |  |
|  |  |  | Weighted median | **0.0846** | **0.0058** | **<0.001** |  |  |  |
| Type II diabetes | HbA1c | 115 | IVW | **0.0595** | **0.0050** | **<0.001** | **796.41** | **<0.001** | 0.960 |
|  |  |  | IVW (multiplicative random effects) | **0.0595** | **0.0050** | **<0.001** |  |  |  |
|  |  |  | MR Egger | **0.0590** | **0.0114** | **<0.001** | **796.39** | **<0.001** |  |
|  |  |  | MR Egger (bootstrap) | **0.0534** | **0.0050** | **<0.001** |  |  |  |
|  |  |  | Weighted median | **0.0499** | **0.0037** | **<0.001** |  |  |  |
| Fasting glucose | Type II diabetes | 55 | IVW | **1.7323** | **0.3246** | **<0.001** | **2211.53** | **<0.001** | 0.068 |
|  |  |  | IVW (multiplicative random effects) | **1.7323** | **0.3246** | **<0.001** |  |  |  |
|  |  |  | MR Egger | 0.8540 | 0.5689 | 0.139 | **2075.93** | **<0.001** |  |
|  |  |  | MR Egger (bootstrap) | **0.9670** | **0.1123** | **<0.001** |  |  |  |
|  |  |  | Weighted median | **1.0908** | **0.1128** | **<0.001** |  |  |  |
| HbA1c | Type II diabetes | 56 | IVW | **1.3678** | **0.4950** | **0.006** | **2527.34** | **<0.001** | **0.041** |
|  |  |  | IVW (multiplicative random effects) | **1.3678** | **0.4950** | **0.006** |  |  |  |
|  |  |  | MR Egger | -0.0652 | 0.8353 | 0.938 | **2336.99** | **<0.001** |  |
|  |  |  | MR Egger (bootstrap) | **0.7620** | **0.2974** | **0.009** |  |  |  |
|  |  |  | Weighted median | **0.3176** | **0.1296** | **0.014** |  |  |  |
|  |  |  | MR-PRESSO | **0.8416** | **0.1735** | **<0.001** |  |  |  |

HbA1c: glycated hemoglobin; MR: Mendelian randomization; SNP: single nucleotide polymorphisms; IVW: inverse variance weighted; MR Egger: Egger's regression for Mendelian randomization; MR-PRESSO: Mendelian Randomization Pleiotropy RESidual Sum and Outlier.

**Table S3-S5 were in the “Supplementary datasets” file.**

**Table S6. Causal associations between fasting glucose, HbA1c, type II diabetes and kidney cancer utilizing two-sample MR.**

| **Exposures** | **Outcomes** | **Consortium of outcomes** | **SNPs** | **MR methods** | **Beta** | **Standard error** | **P value** | **Q_heterogeneity_** | **P_heterogeneity_** | **P_pleiotropy_** |
| --- | --- | --- | --- | --- | --- | --- | --- | --- | --- | --- |
| Fasting glucose | Kidney cancer | FinnGen Biobank | 63 | IVW | 0.3100 | 0.2967 | 0.296 | 63.66 | 0.418 | 0.265 |
|  |  |  |  | IVW (multiplicative random effects) | 0.3100 | 0.2967 | 0.296 |  |  |  |
|  |  |  |  | MR Egger | -0.1884 | 0.5330 | 0.725 | 62.37 | 0.427 |  |
|  |  |  |  | MR Egger (bootstrap) | -0.0103 | 0.4774 | 0.489 |  |  |  |
|  |  |  |  | Weighted median | 0.2085 | 0.4355 | 0.632 |  |  |  |
|  |  | UK Biobank | 30 | IVW | -0.0007 | 0.0010 | 0.451 | 23.24 | 0.765 | 0.720 |
|  |  |  |  | IVW (multiplicative random effects) | -0.0007 | 0.0009 | 0.400 |  |  |  |
|  |  |  |  | MR Egger | -0.0002 | 0.0019 | 0.932 | 23.11 | 0.728 |  |
|  |  |  |  | MR Egger (bootstrap) | -0.00003 | 0.0019 | 0.500 |  |  |  |
|  |  |  |  | Weighted median | -0.0007 | 0.0015 | 0.639 |  |  |  |
| HbA1c | Kidney cancer | FinnGen Biobank | 70 | IVW | 0.1742 | 0.4209 | 0.679 | 61.84 | 0.717 | 0.492 |
|  |  |  |  | IVW (multiplicative random effects) | 0.1742 | 0.3984 | 0.662 |  |  |  |
|  |  |  |  | MR Egger | -0.3062 | 0.8126 | 0.708 | 61.37 | 0.702 |  |
|  |  |  |  | MR Egger (bootstrap) | -1.1346 | 0.9493 | 0.109 |  |  |  |
|  |  |  |  | Weighted median | 0.4902 | 0.6501 | 0.451 |  |  |  |
|  |  | UK Biobank | 24 | IVW | -0.0013 | 0.0017 | 0.451 | 20.74 | 0.597 | **0.005** |
|  |  |  |  | IVW (multiplicative random effects) | -0.0013 | 0.0016 | 0.427 |  |  |  |
|  |  |  |  | MR Egger | **-0.0127** | **0.0041** | **0.005** | 11.13 | 0.973 |  |
|  |  |  |  | MR Egger (bootstrap) | **-0.0124** | **0.0045** | **0.002** |  |  |  |
|  |  |  |  | Weighted median | -0.0005 | 0.0025 | 0.851 |  |  |  |
| Type II diabetes | Kidney cancer | FinnGen Biobank | 114 | IVW | 0.0367 | 0.0779 | 0.638 | **142.25** | **0.033** | 0.759 |
|  |  |  |  | IVW (multiplicative random effects) | 0.0367 | 0.0779 | 0.638 |  |  |  |
|  |  |  |  | MR Egger | -0.0151 | 0.1856 | 0.935 | **142.13** | **0.029** |  |
|  |  |  |  | MR Egger (bootstrap) | -0.0123 | 0.1531 | 0.473 |  |  |  |
|  |  |  |  | Weighted median | -0.0102 | 0.1307 | 0.938 |  |  |  |
|  |  | UK Biobank | 54 | IVW | 0.0001 | 0.0002 | 0.589 | 44.69 | 0.785 | 0.779 |
|  |  |  |  | IVW (multiplicative random effects) | 0.0001 | 0.0002 | 0.556 |  |  |  |
|  |  |  |  | MR Egger | 0.0002 | 0.0004 | 0.606 | 44.61 | 0.757 |  |
|  |  |  |  | MR Egger (bootstrap) | 0.0002 | 0.0004 | 0.274 |  |  |  |
|  |  |  |  | Weighted median | -0.000008 | 0.0004 | 0.979 |  |  |  |

HbA1c: glycated hemoglobin; MR: Mendelian randomization; SNP: single nucleotide polymorphisms; IVW: inverse variance weighted; MR Egger: Egger's regression for Mendelian randomization.

**Table S7. Causal associations between fasting glucose, HbA1c, type II diabetes and bladder cancer utilizing two-sample MR.**

| **Exposures** | **Outcomes** | **Consortium of outcomes** | **SNPs** | **MR methods** | **Beta** | **Standard error** | **P value** | **Q_heterogeneity_** | **P_heterogeneity_** | **P_pleiotropy_** |
| --- | --- | --- | --- | --- | --- | --- | --- | --- | --- | --- |
| Fasting glucose | Bladder cancer | UK Biobank | 63 | IVW | 0.0009 | 0.0009 | 0.287 | 59.82 | 0.555 | 0.388 |
|  |  |  |  | IVW (multiplicative random effects) | 0.0009 | 0.0008 | 0.279 |  |  |  |
|  |  |  |  | MR Egger | -0.0002 | 0.0016 | 0.888 | 59.07 | 0.546 |  |
|  |  |  |  | MR Egger (bootstrap) | 0.0013 | 0.0015 | 0.189 |  |  |  |
|  |  |  |  | Weighted median | 0.0005 | 0.0013 | 0.734 |  |  |  |
|  |  | FinnGen Biobank | 63 | IVW | 0.2419 | 0.2732 | 0.376 | 50.95 | 0.841 | 0.198 |
|  |  |  |  | IVW (multiplicative random effects) | 0.2419 | 0.2476 | 0.329 |  |  |  |
|  |  |  |  | MR Egger | 0.7738 | 0.4918 | 0.121 | 49.26 | 0.860 |  |
|  |  |  |  | MR Egger (bootstrap) | 0.6909 | 0.4557 | 0.069 |  |  |  |
|  |  |  |  | Weighted median | 0.2187 | 0.4373 | 0.617 |  |  |  |
| HbA1c | Bladder cancer | UK Biobank | 67 | IVW | 0.0020 | 0.0013 | 0.112 | 76.54 | 0.176 | **0.012** |
|  |  |  |  | IVW (multiplicative random effects) | 0.0020 | 0.0013 | 0.112 |  |  |  |
|  |  |  |  | MR Egger | **0.0066** | **0.0022** | **0.003** | 69.51 | 0.328 |  |
|  |  |  |  | MR Egger (bootstrap) | **0.0055** | **0.0024** | **0.009** |  |  |  |
|  |  |  |  | Weighted median | 0.0036 | 0.0019 | 0.053 |  |  |  |
|  |  | FinnGen Biobank | 70 | IVW | 0.5856 | 0.4893 | 0.231 | **107.46** | **0.002** | 0.622 |
|  |  |  |  | IVW (multiplicative random effects) | 0.5856 | 0.4893 | 0.231 |  |  |  |
|  |  |  |  | MR Egger | 0.9877 | 0.9499 | 0.302 | **107.07** | **0.002** |  |
|  |  |  |  | MR Egger (bootstrap) | 0.8251 | 0.8615 | 0.169 |  |  |  |
|  |  |  |  | Weighted median | 1.0819 | 0.6930 | 0.118 |  |  |  |
| Type II diabetes | Bladder cancer | UK Biobank | 113 | IVW | 0.0001 | 0.0002 | 0.752 | 120.75 | 0.270 | 0.100 |
|  |  |  |  | IVW (multiplicative random effects) | 0.0001 | 0.0002 | 0.752 |  |  |  |
|  |  |  |  | MR Egger | 0.0008 | 0.0005 | 0.106 | 117.83 | 0.311 |  |
|  |  |  |  | MR Egger (bootstrap) | **0.0008** | **0.0004** | **0.030** |  |  |  |
|  |  |  |  | Weighted median | 0.0005 | 0.0003 | 0.149 |  |  |  |
|  |  | FinnGen Biobank | 114 | IVW | 0.0129 | 0.0683 | 0.850 | 125.95 | 0.191 | 0.557 |
|  |  |  |  | IVW (multiplicative random effects) | 0.0129 | 0.0683 | 0.850 |  |  |  |
|  |  |  |  | MR Egger | 0.0997 | 0.1625 | 0.541 | 125.56 | 0.180 |  |
|  |  |  |  | MR Egger (bootstrap) | -0.0194 | 0.1405 | 0.445 |  |  |  |
|  |  |  |  | Weighted median | -0.1056 | 0.1019 | 0.300 |  |  |  |

HbA1c: glycated hemoglobin; MR: Mendelian randomization; SNP: single nucleotide polymorphisms; IVW: inverse variance weighted; MR Egger: Egger's regression for Mendelian randomization.

**Table S8. Causal associations between fasting glucose, HbA1c, type II diabetes and prostate cancer utilizing two-sample MR.**

| **Exposures** | **Outcomes** | **Consortium of outcomes** | **SNPs** | **MR methods** | **Beta** | **Standard error** | **P value** | **Q_heterogeneity_** | **P_heterogeneity_** | **P_pleiotropy_** |
| --- | --- | --- | --- | --- | --- | --- | --- | --- | --- | --- |
| Fasting glucose | Prostate cancer | UK Biobank | 63 | IVW | -0.0032 | 0.0062 | 0.609 | **114.89** | **<0.001** | 0.273 |
|  |  |  |  | IVW (multiplicative random effects) | -0.0032 | 0.0062 | 0.609 |  |  |  |
|  |  |  |  | MR Egger | 0.0073 | 0.0113 | 0.522 | **112.63** | **<0.001** |  |
|  |  |  |  | MR Egger (bootstrap) | 0.0105 | 0.0080 | 0.097 |  |  |  |
|  |  |  |  | Weighted median | 0.0001 | 0.0070 | 0.991 |  |  |  |
|  |  | PRACTICAL | 63 | IVW | -0.1219 | 0.1188 | 0.305 | **316.46** | **<0.001** | 0.497 |
|  |  |  |  | IVW (multiplicative random effects) | -0.1219 | 0.1188 | 0.305 |  |  |  |
|  |  |  |  | MR Egger | 0.0039 | 0.2193 | 0.986 | **314.05** | **<0.001** |  |
|  |  |  |  | MR Egger (bootstrap) | -0.1165 | 0.0994 | 0.124 |  |  |  |
|  |  |  |  | Weighted median | -0.1051 | 0.0835 | 0.208 |  |  |  |
|  |  | FinnGen Biobank | 63 | IVW | -0.0206 | 0.1441 | 0.886 | 76.41 | 0.103 | 0.440 |
|  |  |  |  | IVW (multiplicative random effects) | -0.0206 | 0.1441 | 0.886 |  |  |  |
|  |  |  |  | MR Egger | -0.1891 | 0.2604 | 0.471 | 75.66 | 0.098 |  |
|  |  |  |  | MR Egger (bootstrap) | -0.2506 | 0.2058 | 0.104 |  |  |  |
|  |  |  |  | Weighted median | -0.2856 | 0.1949 | 0.143 |  |  |  |
| HbA1c | Prostate cancer | UK Biobank | 67 | IVW | -0.0127 | 0.0086 | 0.141 | **122.61** | **<0.001** | 0.864 |
|  |  |  |  | IVW (multiplicative random effects) | -0.0127 | 0.0086 | 0.141 |  |  |  |
|  |  |  |  | MR Egger | -0.0105 | 0.0154 | 0.495 | **122.55** | **<0.001** |  |
|  |  |  |  | MR Egger (bootstrap) | 0.0007 | 0.0131 | 0.482 |  |  |  |
|  |  |  |  | Weighted median | -0.0125 | 0.0109 | 0.252 |  |  |  |
|  |  | PRACTICAL | 66 | IVW | -0.0977 | 0.1310 | 0.456 | **199.88** | **<0.001** | 0.485 |
|  |  |  |  | IVW (multiplicative random effects) | -0.0977 | 0.1310 | 0.456 |  |  |  |
|  |  |  |  | MR Egger | 0.0404 | 0.2367 | 0.865 | **198.36** | **<0.001** |  |
|  |  |  |  | MR Egger (bootstrap) | 0.1682 | 0.1545 | 0.143 |  |  |  |
|  |  |  |  | Weighted median | -0.1199 | 0.1263 | 0.343 |  |  |  |
|  |  | FinnGen Biobank | 70 | IVW | -0.0903 | 0.1903 | 0.635 | 71.89 | 0.382 | 0.928 |
|  |  |  |  | IVW (multiplicative random effects) | -0.0903 | 0.1903 | 0.635 |  |  |  |
|  |  |  |  | MR Egger | -0.0616 | 0.3702 | 0.868 | 71.88 | 0.351 |  |
|  |  |  |  | MR Egger (bootstrap) | 0.1990 | 0.4123 | 0.307 |  |  |  |
|  |  |  |  | Weighted median | 0.0153 | 0.3094 | 0.960 |  |  |  |
| Type II diabetes | Prostate cancer | UK Biobank | 113 | IVW | -0.0006 | 0.0013 | 0.653 | **170.54** | **<0.001** | **0.005** |
|  |  |  |  | IVW (multiplicative random effects) | -0.0006 | 0.0013 | 0.653 |  |  |  |
|  |  |  |  | MR Egger | **0.0067** | **0.0029** | **0.021** | **159.01** | **0.002** |  |
|  |  |  |  | MR Egger (bootstrap) | **0.0053** | **0.0023** | **0.008** |  |  |  |
|  |  |  |  | Weighted median | 0.0012 | 0.0017 | 0.470 |  |  |  |
|  |  | PRACTICAL | 113 | IVW | -0.0232 | 0.0206 | 0.261 | **332.57** | **<0.001** | 0.307 |
|  |  |  |  | IVW (multiplicative random effects) | -0.0232 | 0.0206 | 0.261 |  |  |  |
|  |  |  |  | MR Egger | 0.0200 | 0.0468 | 0.670 | **329.44** | **<0.001** |  |
|  |  |  |  | MR Egger (bootstrap) | -0.0027 | 0.0271 | 0.451 |  |  |  |
|  |  |  |  | Weighted median | -0.0003 | 0.0204 | 0.989 |  |  |  |
|  |  | FinnGen Biobank | 114 | IVW | -0.0313 | 0.0308 | 0.308 | 106.09 | 0.664 | 0.784 |
|  |  |  |  | IVW (multiplicative random effects) | -0.0313 | 0.0298 | 0.293 |  |  |  |
|  |  |  |  | MR Egger | -0.0132 | 0.0729 | 0.857 | 106.02 | 0.641 |  |
|  |  |  |  | MR Egger (bootstrap) | -0.0465 | 0.0655 | 0.250 |  |  |  |
|  |  |  |  | Weighted median | 0.0040 | 0.0520 | 0.939 |  |  |  |

HbA1c: glycated hemoglobin; MR: Mendelian randomization; SNP: single nucleotide polymorphisms; PRACTICAL: Prostate Cancer Association Group to Investigate Cancer Associated Alterations in the Genome; IVW: inverse variance weighted; MR Egger: Egger's regression for Mendelian randomization.

**Table S9. Causal associations between fasting glucose, HbA1c, type II diabetes and kidney/ureter stone utilizing two-sample MR.**

| **Exposures** | **Outcomes** | **Consortium of outcomes** | **SNPs** | **MR methods** | **Beta** | **Standard error** | **P value** | **Q_heterogeneity_** | **P_heterogeneity_** | **P_pleiotropy_** |
| --- | --- | --- | --- | --- | --- | --- | --- | --- | --- | --- |
| Fasting glucose | Kidney/Ureter stone | FinnGen Biobank | 63 | IVW | 0.1753 | 0.1444 | 0.225 | 71.86 | 0.184 | 0.507 |
|  |  |  |  | IVW (multiplicative random effects) | 0.1753 | 0.1444 | 0.225 |  |  |  |
|  |  |  |  | MR Egger | 0.0303 | 0.2611 | 0.908 | 71.34 | 0.172 |  |
|  |  |  |  | MR Egger (bootstrap) | 0.0014 | 0.2278 | 0.480 |  |  |  |
|  |  |  |  | Weighted median | 0.0622 | 0.1992 | 0.755 |  |  |  |
|  |  | UK Biobank | 58 | IVW | 0.0009 | 0.0013 | 0.502 | 70.88 | 0.102 | 0.846 |
|  |  |  |  | IVW (multiplicative random effects) | 0.0009 | 0.0013 | 0.502 |  |  |  |
|  |  |  |  | MR Egger | 0.0005 | 0.0024 | 0.837 | 70.83 | 0.088 |  |
|  |  |  |  | MR Egger (bootstrap) | -0.0003 | 0.0020 | 0.453 |  |  |  |
|  |  |  |  | Weighted median | 0.0001 | 0.0018 | 0.969 |  |  |  |
| HbA1c | Kidney/Ureter stone | FinnGen Biobank | 70 | IVW | 0.0047 | 0.1997 | 0.981 | 73.96 | 0.320 | 0.144 |
|  |  |  |  | IVW (multiplicative random effects) | 0.0047 | 0.1997 | 0.981 |  |  |  |
|  |  |  |  | MR Egger | 0.4892 | 0.3827 | 0.206 | 71.65 | 0.358 |  |
|  |  |  |  | MR Egger (bootstrap) | 0.0807 | 0.4041 | 0.433 |  |  |  |
|  |  |  |  | Weighted median | -0.2690 | 0.3205 | 0.401 |  |  |  |
|  |  | UK Biobank | 64 | IVW | 0.0016 | 0.0017 | 0.347 | 69.75 | 0.261 | 0.473 |
|  |  |  |  | IVW (multiplicative random effects) | 0.0016 | 0.0017 | 0.347 |  |  |  |
|  |  |  |  | MR Egger | -0.0003 | 0.0031 | 0.933 | 69.17 | 0.248 |  |
|  |  |  |  | MR Egger (bootstrap) | -0.0019 | 0.0034 | 0.280 |  |  |  |
|  |  |  |  | Weighted median | 0.0024 | 0.0027 | 0.371 |  |  |  |
| Type II diabetes | Kidney/Ureter stone | FinnGen Biobank | 114 | IVW | 0.0168 | 0.0439 | 0.702 | **214.98** | **<0.001** | 0.582 |
|  |  |  |  | IVW (multiplicative random effects) | 0.0168 | 0.0439 | 0.702 |  |  |  |
|  |  |  |  | MR Egger | -0.0356 | 0.1045 | 0.734 | **214.39** | **<0.001** |  |
|  |  |  |  | MR Egger (bootstrap) | 0.0217 | 0.0733 | 0.383 |  |  |  |
|  |  |  |  | Weighted median | -0.0471 | 0.0546 | 0.389 |  |  |  |
|  |  | UK Biobank | 106 | IVW | 0.0001 | 0.0003 | 0.865 | **153.18** | **0.002** | 0.977 |
|  |  |  |  | IVW (multiplicative random effects) | 0.0001 | 0.0003 | 0.865 |  |  |  |
|  |  |  |  | MR Egger | 0.0001 | 0.0008 | 0.920 | **153.18** | **0.001** |  |
|  |  |  |  | MR Egger (bootstrap) | 0.0003 | 0.0006 | 0.307 |  |  |  |
|  |  |  |  | Weighted median | 0.0001 | 0.0005 | 0.888 |  |  |  |

HbA1c: glycated hemoglobin; MR: Mendelian randomization; SNP: single nucleotide polymorphisms; IVW: inverse variance weighted; MR Egger: Egger's regression for Mendelian randomization.

**Table S10. Causal associations between fasting glucose, HbA1c, type II diabetes and urinary incontinence utilizing two-sample MR.**

| **Exposures** | **Outcomes** | **Consortium of outcomes** | **SNPs** | **MR methods** | **Beta** | **Standard error** | **P value** | **Q_heterogeneity_** | **P_heterogeneity_** | **P_pleiotropy_** |
| --- | --- | --- | --- | --- | --- | --- | --- | --- | --- | --- |
| Fasting glucose | Urinary incontinence | FinnGen Biobank | 63 | IVW | -0.1956 | 0.2492 | 0.432 | 61.69 | 0.487 | 0.198 |
|  |  |  |  | IVW (multiplicative random effects) | -0.1956 | 0.2486 | 0.431 |  |  |  |
|  |  |  |  | MR Egger | -0.6816 | 0.4487 | 0.134 | 60.00 | 0.512 |  |
|  |  |  |  | MR Egger (bootstrap) | -0.4294 | 0.4079 | 0.149 |  |  |  |
|  |  |  |  | Weighted median | -0.3637 | 0.3729 | 0.329 |  |  |  |
|  |  | UK Biobank | 45 | IVW | 0.0010 | 0.0008 | 0.223 | 40.98 | 0.602 | 0.993 |
|  |  |  |  | IVW (multiplicative random effects) | 0.0010 | 0.0008 | 0.206 |  |  |  |
|  |  |  |  | MR Egger | 0.0010 | 0.0015 | 0.477 | 40.98 | 0.559 |  |
|  |  |  |  | MR Egger (bootstrap) | 0.0010 | 0.0015 | 0.245 |  |  |  |
|  |  |  |  | Weighted median | 0.0017 | 0.0013 | 0.176 |  |  |  |
| HbA1c | Urinary incontinence | FinnGen Biobank | 70 | IVW | 0.3466 | 0.3753 | 0.356 | 76.66 | 0.246 | 0.587 |
|  |  |  |  | IVW (multiplicative random effects) | 0.3466 | 0.3753 | 0.356 |  |  |  |
|  |  |  |  | MR Egger | 0.0097 | 0.7233 | 0.989 | 76.32 | 0.229 |  |
|  |  |  |  | MR Egger (bootstrap) | -0.5674 | 0.7571 | 0.234 |  |  |  |
|  |  |  |  | Weighted median | 0.3179 | 0.5859 | 0.587 |  |  |  |
|  |  | UK Biobank | 46 | IVW | 0.0020 | 0.0014 | 0.173 | 47.65 | 0.365 | 0.060 |
|  |  |  |  | IVW (multiplicative random effects) | 0.0020 | 0.0014 | 0.173 |  |  |  |
|  |  |  |  | MR Egger | **0.0075** | **0.0032** | **0.023** | 43.92 | 0.475 |  |
|  |  |  |  | MR Egger (bootstrap) | **0.0066** | **0.0034** | **0.022** |  |  |  |
|  |  |  |  | Weighted median | 0.0025 | 0.0023 | 0.270 |  |  |  |
| Type II diabetes | Urinary incontinence | FinnGen Biobank | 114 | IVW | 0.0224 | 0.0622 | 0.719 | 126.03 | 0.190 | 0.907 |
|  |  |  |  | IVW (multiplicative random effects) | 0.0224 | 0.0622 | 0.719 |  |  |  |
|  |  |  |  | MR Egger | 0.0381 | 0.1478 | 0.797 | 126.02 | 0.173 |  |
|  |  |  |  | MR Egger (bootstrap) | 0.0299 | 0.1290 | 0.405 |  |  |  |
|  |  |  |  | Weighted median | 0.0269 | 0.1041 | 0.796 |  |  |  |
|  |  | UK Biobank | 82 | IVW | 0.0001 | 0.0002 | 0.720 | 85.09 | 0.356 | 0.872 |
|  |  |  |  | IVW (multiplicative random effects) | 0.0001 | 0.0002 | 0.720 |  |  |  |
|  |  |  |  | MR Egger | 0.00001 | 0.0005 | 0.983 | 85.06 | 0.328 |  |
|  |  |  |  | MR Egger (bootstrap) | 0.0001 | 0.0004 | 0.424 |  |  |  |
|  |  |  |  | Weighted median | -0.0002 | 0.0004 | 0.589 |  |  |  |

HbA1c: glycated hemoglobin; MR: Mendelian randomization; SNP: single nucleotide polymorphisms; IVW: inverse variance weighted; MR Egger: Egger's regression for Mendelian randomization.

**Table S11. Causal associations between fasting glucose, HbA1c, type II diabetes and BPH utilizing two-sample MR.**

| **Exposures** | **Outcomes** | **Consortium of outcomes** | **SNPs** | **MR methods** | **Beta** | **Standard error** | **P value** | **Q_heterogeneity_** | **P_heterogeneity_** | **P_pleiotropy_** |
| --- | --- | --- | --- | --- | --- | --- | --- | --- | --- | --- |
| Fasting glucose | BPH | FinnGen Biobank | 63 | IVW | 0.0405 | 0.1013 | 0.689 | 57.98 | 0.621 | 0.636 |
|  |  |  |  | IVW (multiplicative random effects) | 0.0405 | 0.0980 | 0.679 |  |  |  |
|  |  |  |  | MR Egger | 0.1128 | 0.1823 | 0.539 | 57.76 | 0.594 |  |
|  |  |  |  | MR Egger (bootstrap) | 0.1318 | 0.1670 | 0.213 |  |  |  |
|  |  |  |  | Weighted median | 0.0523 | 0.1544 | 0.735 |  |  |  |
| HbA1c | BPH | FinnGen Biobank | 70 | IVW | 0.1039 | 0.1471 | 0.480 | 70.47 | 0.428 | 0.299 |
|  |  |  |  | IVW (multiplicative random effects) | 0.1039 | 0.1471 | 0.480 |  |  |  |
|  |  |  |  | MR Egger | 0.3587 | 0.2844 | 0.212 | 69.35 | 0.432 |  |
|  |  |  |  | MR Egger (bootstrap) | 0.1286 | 0.3168 | 0.342 |  |  |  |
|  |  |  |  | Weighted median | 0.4052 | 0.2220 | 0.068 |  |  |  |
| Type II diabetes | BPH | FinnGen Biobank | 114 | IVW | -0.0121 | 0.0271 | 0.656 | **143.83** | **0.027** | 0.603 |
|  |  |  |  | IVW (multiplicative random effects) | -0.0121 | 0.0271 | 0.656 |  |  |  |
|  |  |  |  | MR Egger | 0.0184 | 0.0645 | 0.776 | **143.49** | **0.024** |  |
|  |  |  |  | MR Egger (bootstrap) | 0.0197 | 0.0537 | 0.374 |  |  |  |
|  |  |  |  | Weighted median | 0.0182 | 0.0382 | 0.634 |  |  |  |

HbA1c: glycated hemoglobin; BPH: benign prostatic hyperplasia; MR: Mendelian randomization; SNP: single nucleotide polymorphisms; IVW: inverse variance weighted; MR Egger: Egger's regression for Mendelian randomization.

**Table S12. Causal associations between fasting glucose, HbA1c, type II diabetes and erectile dysfunction utilizing two-sample MR.**

| **Exposures** | **Outcomes** | **Consortium of outcomes** | **SNPs** | **MR methods** | **Beta** | **Standard error** | **P value** | **Q_heterogeneity_** | **P_heterogeneity_** | **P_pleiotropy_** |
| --- | --- | --- | --- | --- | --- | --- | --- | --- | --- | --- |
| Fasting glucose | Erectile dysfunction | FinnGen Biobank | 63 | IVW | -0.2193 | 0.3014 | 0.467 | 77.54 | 0.088 | 0.556 |
|  |  |  |  | IVW (multiplicative random effects) | -0.2193 | 0.3014 | 0.467 |  |  |  |
|  |  |  |  | MR Egger | 0.0490 | 0.5454 | 0.929 | 77.10 | 0.080 |  |
|  |  |  |  | MR Egger (bootstrap) | -0.2957 | 0.4428 | 0.248 |  |  |  |
|  |  |  |  | Weighted median | -0.3226 | 0.4152 | 0.437 |  |  |  |
|  |  | - | 64 | IVW | 0.1079 | 0.1397 | 0.440 | 81.26 | 0.061 | 0.480 |
|  |  |  |  | IVW (multiplicative random effects) | 0.1079 | 0.1397 | 0.440 |  |  |  |
|  |  |  |  | MR Egger | 0.2587 | 0.2544 | 0.313 | 80.61 | 0.056 |  |
|  |  |  |  | MR Egger (bootstrap) | 0.0740 | 0.2141 | 0.381 |  |  |  |
|  |  |  |  | Weighted median | 0.1255 | 0.1821 | 0.491 |  |  |  |
| HbA1c | Erectile dysfunction | FinnGen Biobank | 70 | IVW | -0.2724 | 0.3873 | 0.482 | 56.40 | 0.862 | 0.113 |
|  |  |  |  | IVW (multiplicative random effects) | -0.2724 | 0.3502 | 0.437 |  |  |  |
|  |  |  |  | MR Egger | -1.3040 | 0.7498 | 0.087 | 53.81 | 0.895 |  |
|  |  |  |  | MR Egger (bootstrap) | -0.3976 | 0.8344 | 0.324 |  |  |  |
|  |  |  |  | Weighted median | -0.6770 | 0.5744 | 0.239 |  |  |  |
|  |  | - | 72 | IVW | -0.0464 | 0.1673 | 0.782 | 65.82 | 0.652 | 0.920 |
|  |  |  |  | IVW (multiplicative random effects) | -0.0464 | 0.1610 | 0.773 |  |  |  |
|  |  |  |  | MR Egger | -0.0718 | 0.3034 | 0.814 | 65.81 | 0.620 |  |
|  |  |  |  | MR Egger (bootstrap) | 0.0293 | 0.3382 | 0.465 |  |  |  |
|  |  |  |  | Weighted median | -0.1914 | 0.2706 | 0.479 |  |  |  |
| Type II diabetes | Erectile dysfunction | FinnGen Biobank | 114 | IVW | -0.0207 | 0.0722 | 0.775 | **144.76** | **0.024** | **0.001** |
|  |  |  |  | IVW (multiplicative random effects) | -0.0207 | 0.0722 | 0.775 |  |  |  |
|  |  |  |  | MR Egger | **0.4668** | **0.1647** | **0.005** | 132.19 | 0.093 |  |
|  |  |  |  | MR Egger (bootstrap) | **0.3599** | **0.1378** | **0.002** |  |  |  |
|  |  |  |  | Weighted median | 0.1402 | 0.1038 | 0.177 |  |  |  |
|  |  | - | 115 | IVW | **0.1344** | **0.0285** | **<0.001** | 107.32 | 0.658 | 0.956 |
|  |  |  |  | IVW (multiplicative random effects) | **0.1344** | **0.0277** | **<0.001** |  |  |  |
|  |  |  |  | MR Egger | **0.1311** | **0.0651** | **0.046** | 107.32 | 0.633 |  |
|  |  |  |  | MR Egger (bootstrap) | **0.1092** | **0.0618** | **0.038** |  |  |  |
|  |  |  |  | Weighted median | **0.1302** | **0.0582** | **0.025** |  |  |  |

HbA1c: glycated hemoglobin; MR: Mendelian randomization; SNP: single nucleotide polymorphisms; IVW: inverse variance weighted; MR Egger: Egger's regression for Mendelian randomization.

**Table S13. Causal associations between fasting glucose, HbA1c, type II diabetes and female infertility utilizing two-sample MR.**

| **Exposures** | **Outcomes** | **Consortium of outcomes** | **SNPs** | **MR methods** | **Beta** | **Standard error** | **P value** | **Q_heterogeneity_** | **P_heterogeneity_** | **P_pleiotropy_** |
| --- | --- | --- | --- | --- | --- | --- | --- | --- | --- | --- |
| Fasting glucose | Female infertility | FinnGen Biobank | 64 | IVW | 0.1311 | 0.1301 | 0.314 | 75.20 | 0.140 | 0.393 |
|  |  |  |  | IVW (multiplicative random effects) | 0.1311 | 0.1301 | 0.314 |  |  |  |
|  |  |  |  | MR Egger | -0.0362 | 0.2342 | 0.878 | 74.31 | 0.136 |  |
|  |  |  |  | MR Egger (bootstrap) | -0.0735 | 0.1955 | 0.364 |  |  |  |
|  |  |  |  | Weighted median | 0.0589 | 0.1795 | 0.743 |  |  |  |
| HbA1c | Female infertility | FinnGen Biobank | 70 | IVW | 0.3005 | 0.1926 | 0.119 | 87.21 | 0.068 | 0.289 |
|  |  |  |  | IVW (multiplicative random effects) | 0.3005 | 0.1926 | 0.119 |  |  |  |
|  |  |  |  | MR Egger | 0.6401 | 0.3713 | 0.089 | 85.77 | 0.072 |  |
|  |  |  |  | MR Egger (bootstrap) | **0.9778** | **0.3702** | **0.001** |  |  |  |
|  |  |  |  | Weighted median | 0.3487 | 0.2850 | 0.221 |  |  |  |
| Type II diabetes | Female infertility | FinnGen Biobank | 114 | IVW | 0.0254 | 0.0292 | 0.384 | 120.68 | 0.293 | 0.636 |
|  |  |  |  | IVW (multiplicative random effects) | 0.0254 | 0.0292 | 0.384 |  |  |  |
|  |  |  |  | MR Egger | -0.0046 | 0.0697 | 0.948 | 120.44 | 0.276 |  |
|  |  |  |  | MR Egger (bootstrap) | 0.0102 | 0.0604 | 0.442 |  |  |  |
|  |  |  |  | Weighted median | 0.0225 | 0.0544 | 0.679 |  |  |  |

HbA1c: glycated hemoglobin; MR: Mendelian randomization; SNP: single nucleotide polymorphisms; IVW: inverse variance weighted; MR Egger: Egger's regression for Mendelian randomization.

**Table S14. Causal associations between fasting glucose, HbA1c, type II diabetes and male infertility utilizing two-sample MR.**

| **Exposures** | **Outcomes** | **Consortium of outcomes** | **SNPs** | **MR methods** | **Beta** | **Standard error** | **P value** | **Q_heterogeneity_** | **P_heterogeneity_** | **P_pleiotropy_** |
| --- | --- | --- | --- | --- | --- | --- | --- | --- | --- | --- |
| Fasting glucose | Male infertility | FinnGen Biobank | 63 | IVW | -0.2058 | 0.3690 | 0.577 | 69.20 | 0.248 | 0.788 |
|  |  |  |  | IVW (multiplicative random effects) | -0.2058 | 0.3690 | 0.577 |  |  |  |
|  |  |  |  | MR Egger | -0.0555 | 0.6687 | 0.934 | 69.11 | 0.222 |  |
|  |  |  |  | MR Egger (bootstrap) | -0.2000 | 0.5515 | 0.343 |  |  |  |
|  |  |  |  | Weighted median | -0.4881 | 0.5293 | 0.356 |  |  |  |
| HbA1c | Male infertility | FinnGen Biobank | 70 | IVW | -0.5115 | 0.5847 | 0.382 | **93.35** | **0.027** | 0.132 |
|  |  |  |  | IVW (multiplicative random effects) | -0.5115 | 0.5847 | 0.382 |  |  |  |
|  |  |  |  | MR Egger | -1.9763 | 1.1219 | 0.083 | **90.27** | **0.037** |  |
|  |  |  |  | MR Egger (bootstrap) | -1.1856 | 1.0833 | 0.136 |  |  |  |
|  |  |  |  | Weighted median | **-2.4291** | **0.8081** | **0.003** |  |  |  |
| Type II diabetes | Male infertility | FinnGen Biobank | 114 | IVW | **-0.1833** | **0.0829** | **0.027** | 112.91 | 0.485 | **0.038** |
|  |  |  |  | IVW (multiplicative random effects) | **-0.1833** | **0.0829** | **0.027** |  |  |  |
|  |  |  |  | MR Egger | **-0.5595** | **0.1971** | **0.005** | 108.48 | 0.576 |  |
|  |  |  |  | MR Egger (bootstrap) | **-0.5258** | **0.1750** | **0.003** |  |  |  |
|  |  |  |  | Weighted median | **-0.4052** | **0.1403** | **0.004** |  |  |  |

HbA1c: glycated hemoglobin; MR: Mendelian randomization; SNP: single nucleotide polymorphisms; IVW: inverse variance weighted; MR Egger: Egger's regression for Mendelian randomization.

**Table S15. Causal associations between fasting glucose, HbA1c, type II diabetes and abnormal spermatozoa utilizing two-sample MR.**

| **Exposures** | **Outcomes** | **Consortium of outcomes** | **SNPs** | **MR methods** | **Beta** | **Standard error** | **P value** | **Q_heterogeneity_** | **P_heterogeneity_** | **P_pleiotropy_** |
| --- | --- | --- | --- | --- | --- | --- | --- | --- | --- | --- |
| Fasting glucose | Abnormal spermatozoa | FinnGen Biobank | 63 | IVW | 0.2578 | 0.3259 | 0.429 | 71.15 | 0.200 | 0.805 |
|  |  |  |  | IVW (multiplicative random effects) | 0.2578 | 0.3259 | 0.429 |  |  |  |
|  |  |  |  | MR Egger | 0.3799 | 0.5919 | 0.523 | 71.07 | 0.177 |  |
|  |  |  |  | MR Egger (bootstrap) | 0.0733 | 0.5127 | 0.437 |  |  |  |
|  |  |  |  | Weighted median | -0.0727 | 0.4819 | 0.880 |  |  |  |
| HbA1c | Abnormal spermatozoa | FinnGen Biobank | 70 | IVW | -0.1291 | 0.4751 | 0.786 | 82.07 | 0.135 | 0.762 |
|  |  |  |  | IVW (multiplicative random effects) | -0.1291 | 0.4751 | 0.786 |  |  |  |
|  |  |  |  | MR Egger | 0.1104 | 0.9208 | 0.905 | 81.96 | 0.119 |  |
|  |  |  |  | MR Egger (bootstrap) | **1.5703** | **0.9472** | **0.047** |  |  |  |
|  |  |  |  | Weighted median | -0.0501 | 0.6899 | 0.942 |  |  |  |
| Type II diabetes | Abnormal spermatozoa | FinnGen Biobank | 114 | IVW | -0.0086 | 0.0781 | 0.912 | 5.34 | 0.804 | 0.644 |
|  |  |  |  | IVW (multiplicative random effects) | -0.0086 | 0.0781 | 0.912 |  |  |  |
|  |  |  |  | MR Egger | -0.0367 | 0.1859 | 0.844 | 5.11 | 0.746 |  |
|  |  |  |  | MR Egger (bootstrap) | 0.0209 | 0.1534 | 0.444 |  |  |  |
|  |  |  |  | Weighted median | -0.0100 | 0.1260 | 0.937 |  |  |  |

HbA1c: glycated hemoglobin; MR: Mendelian randomization; SNP: single nucleotide polymorphisms; IVW: inverse variance weighted; MR Egger: Egger's regression for Mendelian randomization.

**Table S16. Causal associations between fasting glucose, HbA1c, type II diabetes and polycystic ovary syndrome utilizing two-sample MR.**

| **Exposures** | **Outcomes** | **Consortium of outcomes** | **SNPs** | **MR methods** | **Beta** | **Standard error** | **P value** | **Q_heterogeneity_** | **P_heterogeneity_** | **P_pleiotropy_** |
| --- | --- | --- | --- | --- | --- | --- | --- | --- | --- | --- |
| Fasting glucose | Polycystic ovary syndrome | FinnGen Biobank | 63 | IVW | **0.8586** | **0.3607** | **0.017** | 58.00 | 0.621 | 0.899 |
|  |  |  |  | IVW (multiplicative random effects) | **0.8586** | **0.3489** | **0.014** |  |  |  |
|  |  |  |  | MR Egger | 0.9274 | 0.6491 | 0.158 | 57.98 | 0.586 |  |
|  |  |  |  | MR Egger (bootstrap) | 0.7602 | 0.5957 | 0.107 |  |  |  |
|  |  |  |  | Weighted median | 0.3390 | 0.6224 | 0.586 |  |  |  |
| HbA1c | Polycystic ovary syndrome | FinnGen Biobank | 70 | IVW | **1.1105** | **0.5189** | **0.032** | 69.10 | 0.474 | 0.102 |
|  |  |  |  | IVW (multiplicative random effects) | **1.1105** | **0.5189** | **0.032** |  |  |  |
|  |  |  |  | MR Egger | -0.3078 | 1.0008 | 0.759 | 66.36 | 0.534 |  |
|  |  |  |  | MR Egger (bootstrap) | 1.2552 | 1.1090 | 0.128 |  |  |  |
|  |  |  |  | Weighted median | 0.8467 | 0.7823 | 0.279 |  |  |  |
| Type II diabetes | Polycystic ovary syndrome | FinnGen Biobank | 114 | IVW | **0.1921** | **0.0962** | **0.046** | **143.03** | **0.030** | 0.721 |
|  |  |  |  | IVW (multiplicative random effects) | **0.1921** | **0.0962** | **0.046** |  |  |  |
|  |  |  |  | MR Egger | 0.1175 | 0.2294 | 0.610 | **142.87** | **0.026** |  |
|  |  |  |  | MR Egger (bootstrap) | 0.0645 | 0.1867 | 0.369 |  |  |  |
|  |  |  |  | Weighted median | **0.3061** | **0.1536** | **0.046** |  |  |  |

HbA1c: glycated hemoglobin; MR: Mendelian randomization; SNP: single nucleotide polymorphisms; IVW: inverse variance weighted; MR Egger: Egger's regression for Mendelian randomization.


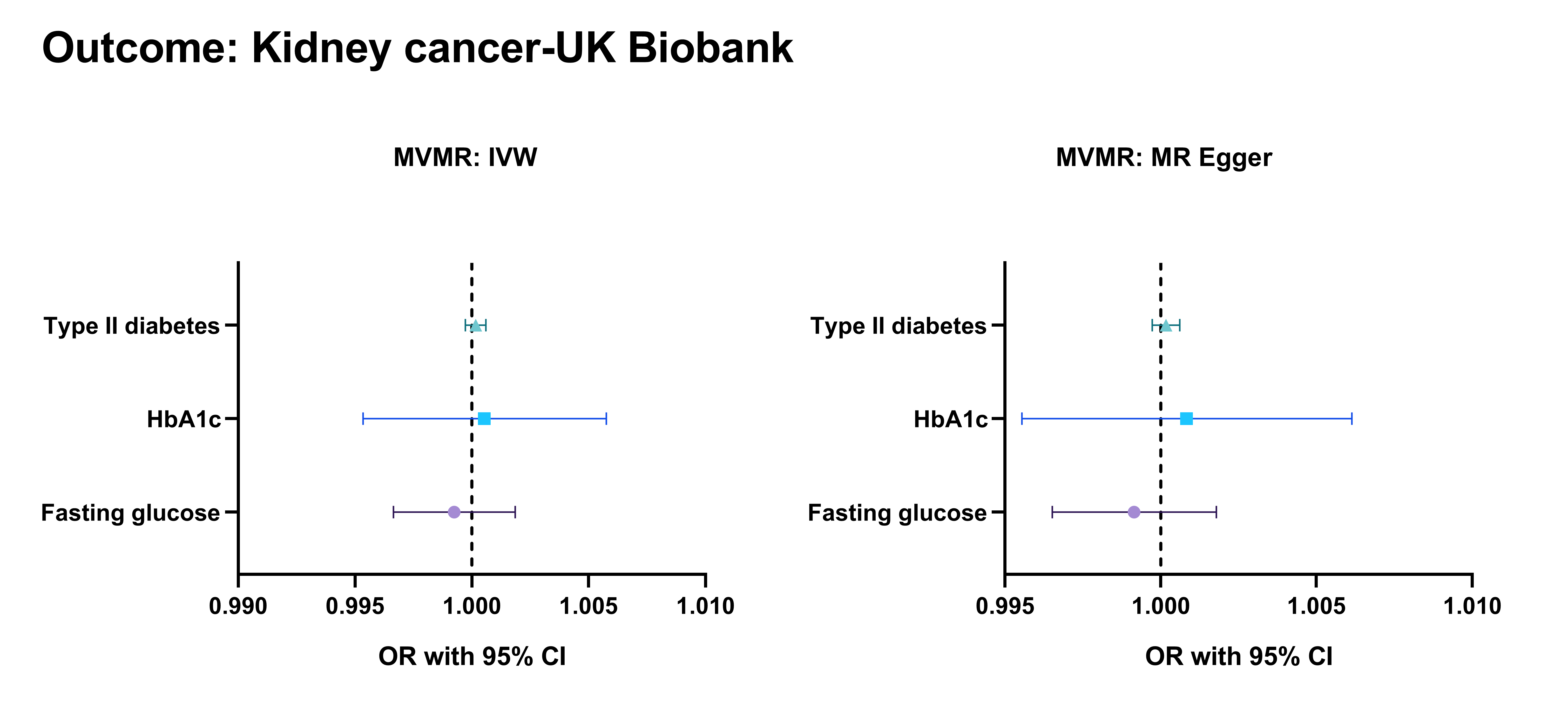


**Figure S1.** MVMR results of the direct effect of three glycemic traits on kidney cancer (UK Biobank) outcome.

MVMR: multivariate Mendelian randomization; HbA1c: glycated hemoglobin; IVW: inverse variance weighted; MR Egger: Egger's regression for Mendelian randomization; OR: odds ratio; 95% CI: 95% confidence interval.


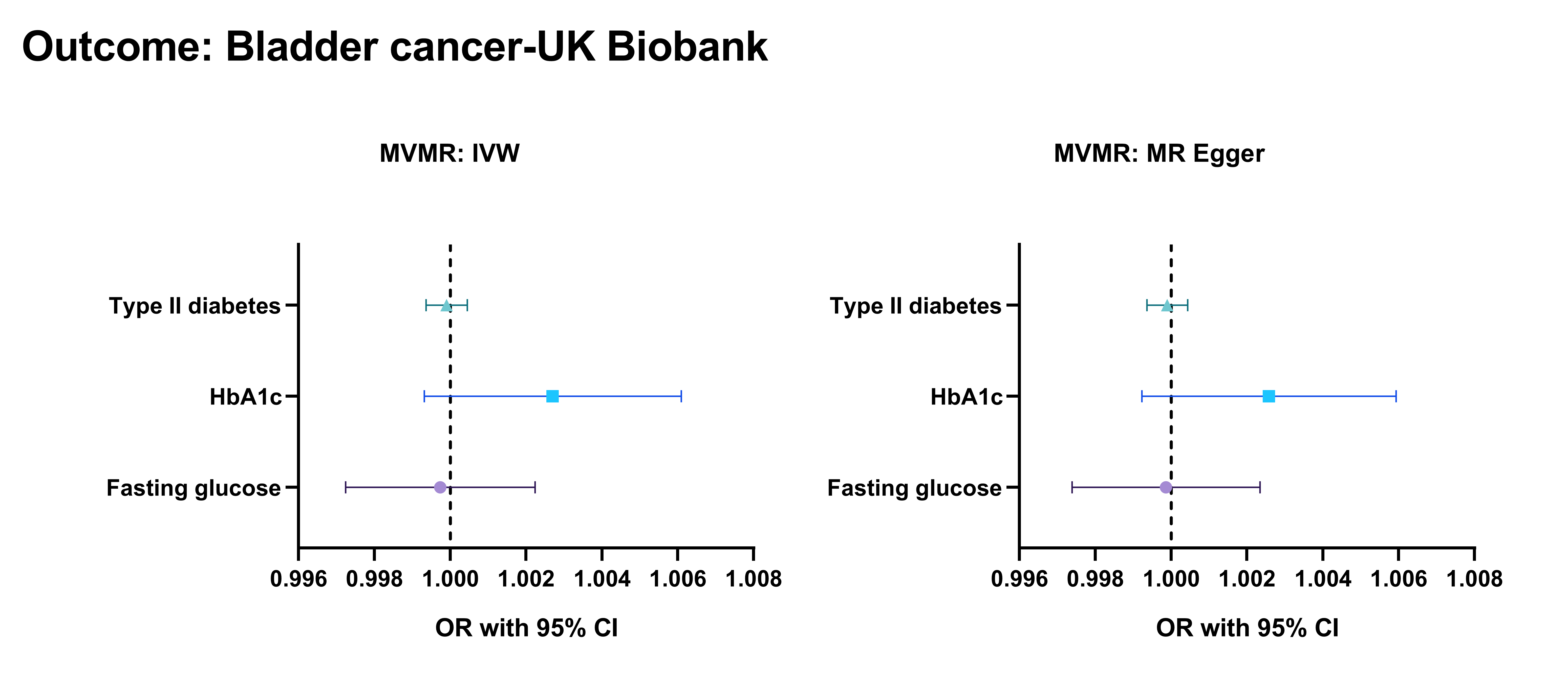


**Figure S2.** MVMR results of the direct effect of three glycemic traits on bladder cancer (UK Biobank) outcome.

MVMR: multivariate Mendelian randomization; HbA1c: glycated hemoglobin; IVW: inverse variance weighted; MR Egger: Egger's regression for Mendelian randomization; OR: odds ratio; 95% CI: 95% confidence interval.


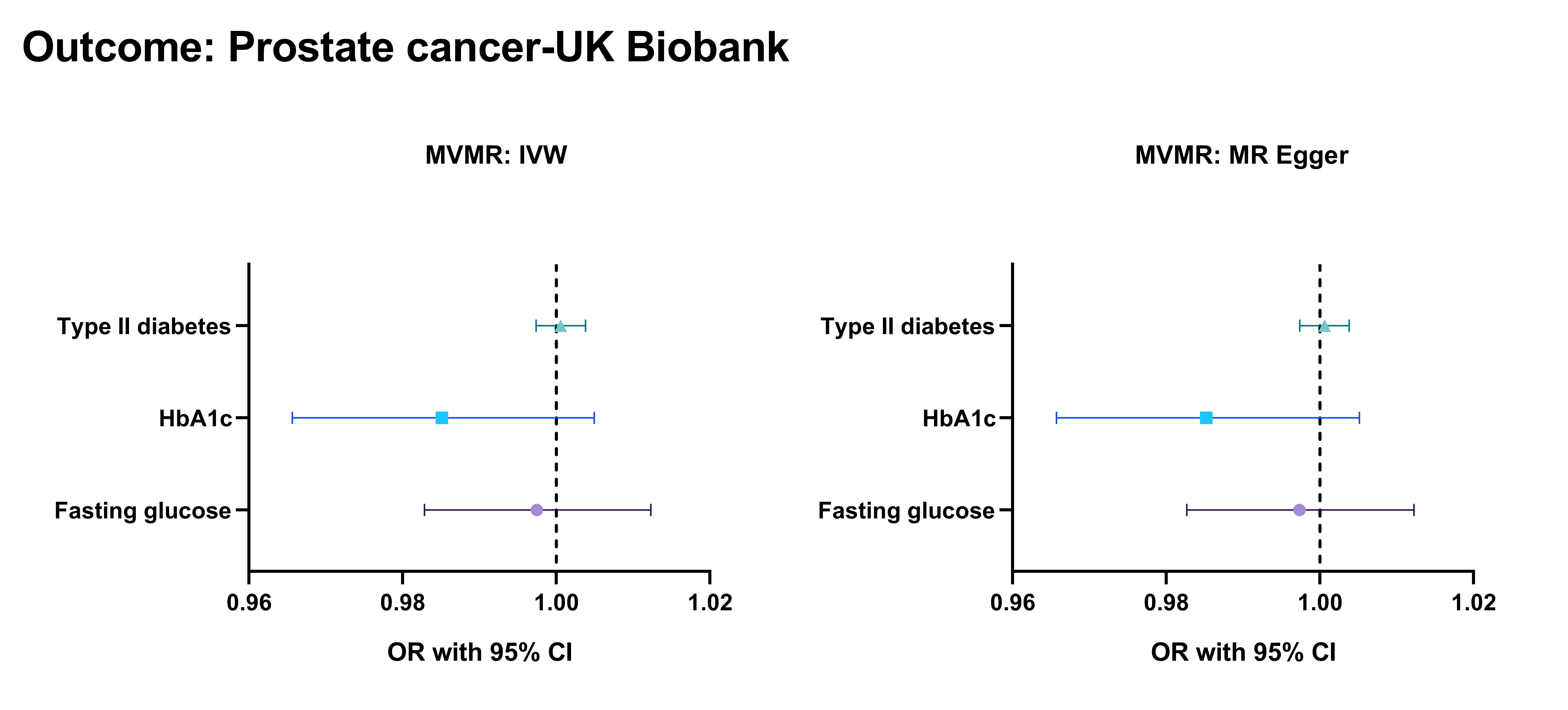


**Figure S3.** MVMR results of the direct effect of three glycemic traits on prostate cancer (UK Biobank) outcome.

MVMR: multivariate Mendelian randomization; HbA1c: glycated hemoglobin; IVW: inverse variance weighted; MR Egger: Egger's regression for Mendelian randomization; OR: odds ratio; 95% CI: 95% confidence interval.


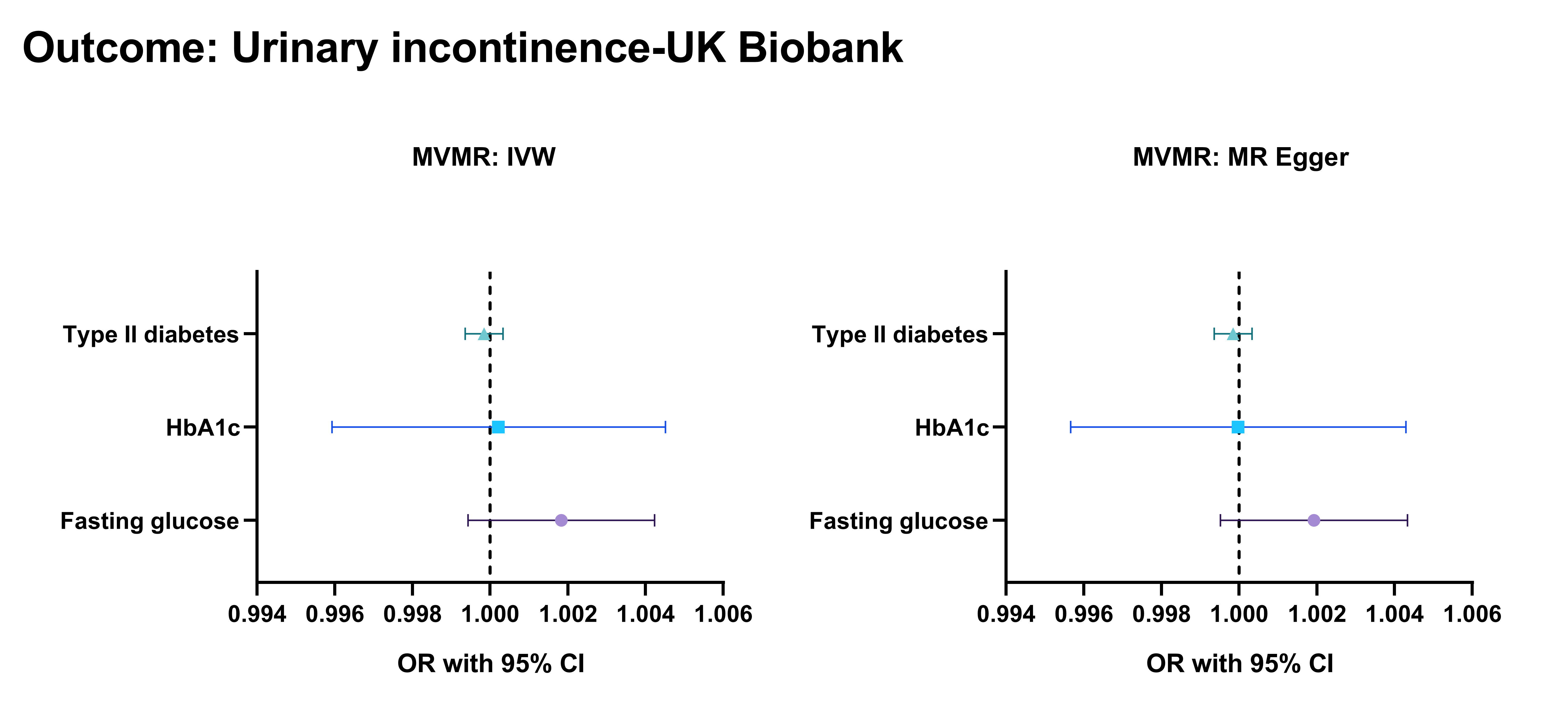


**Figure S4.** MVMR results of the direct effect of three glycemic traits on urinary incontinence (UK Biobank) outcome.

MVMR: multivariate Mendelian randomization; HbA1c: glycated hemoglobin; IVW: inverse variance weighted; MR Egger: Egger's regression for Mendelian randomization; OR: odds ratio; 95% CI: 95% confidence interval.


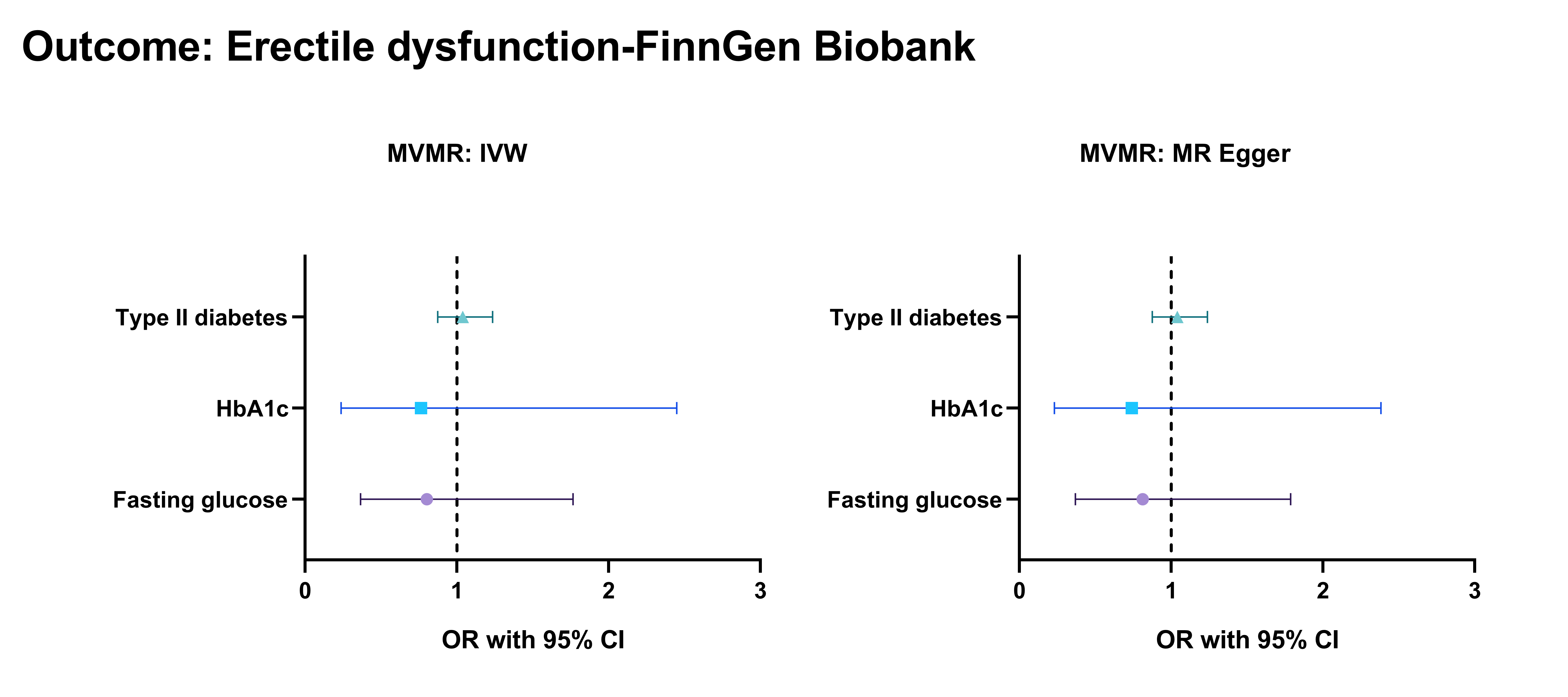


**Figure S5.** MVMR results of the direct effect of three glycemic traits on erectile dysfunction (FinnGen Biobank) outcome.

MVMR: multivariate Mendelian randomization; HbA1c: glycated hemoglobin; IVW: inverse variance weighted; MR Egger: Egger's regression for Mendelian randomization; OR: odds ratio; 95% CI: 95% confidence interval.


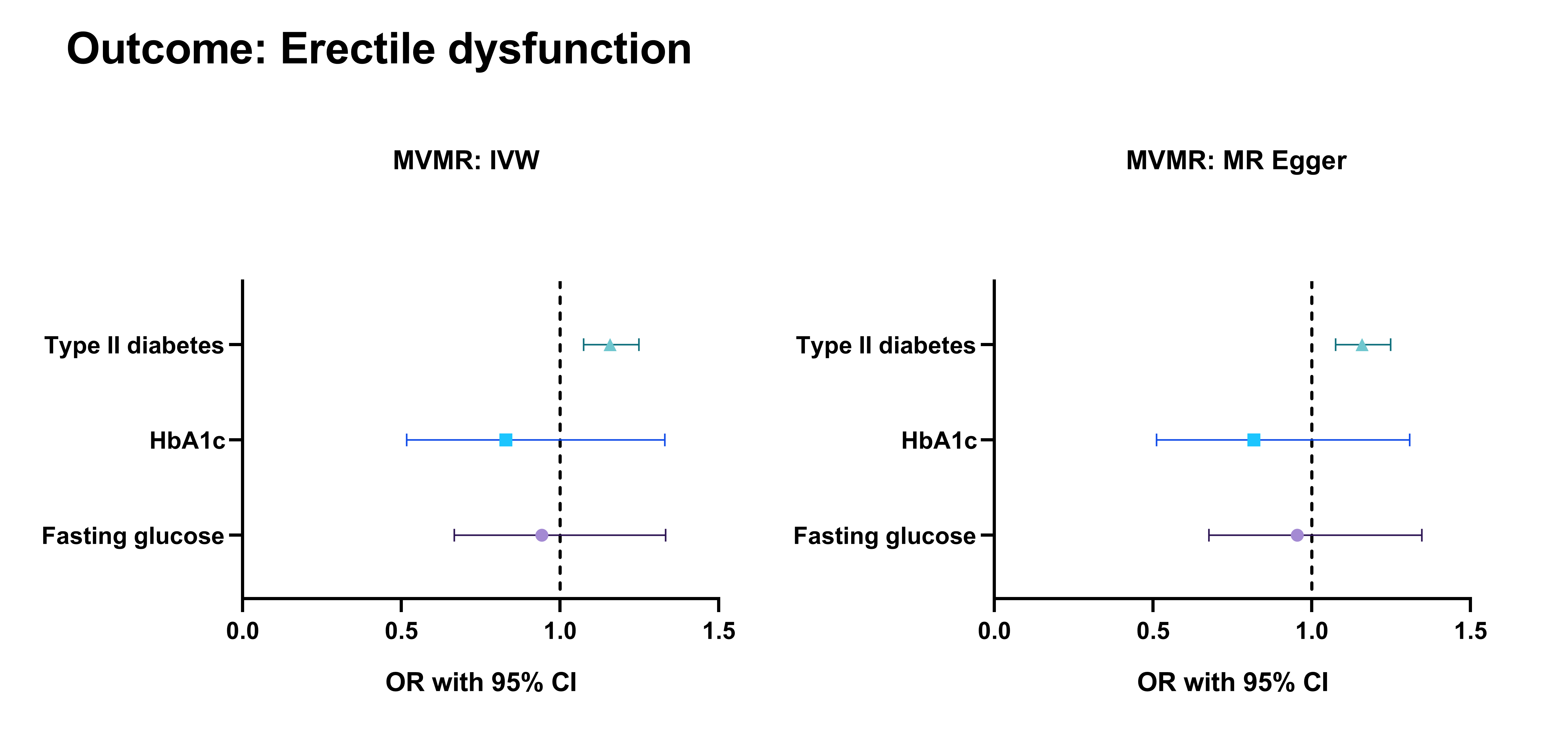


**Figure S6.** MVMR results of the direct effect of three glycemic traits on erectile dysfunction outcome.

MVMR: multivariate Mendelian randomization; HbA1c: glycated hemoglobin; IVW: inverse variance weighted; MR Egger: Egger's regression for Mendelian randomization; OR: odds ratio; 95% CI: 95% confidence interval.


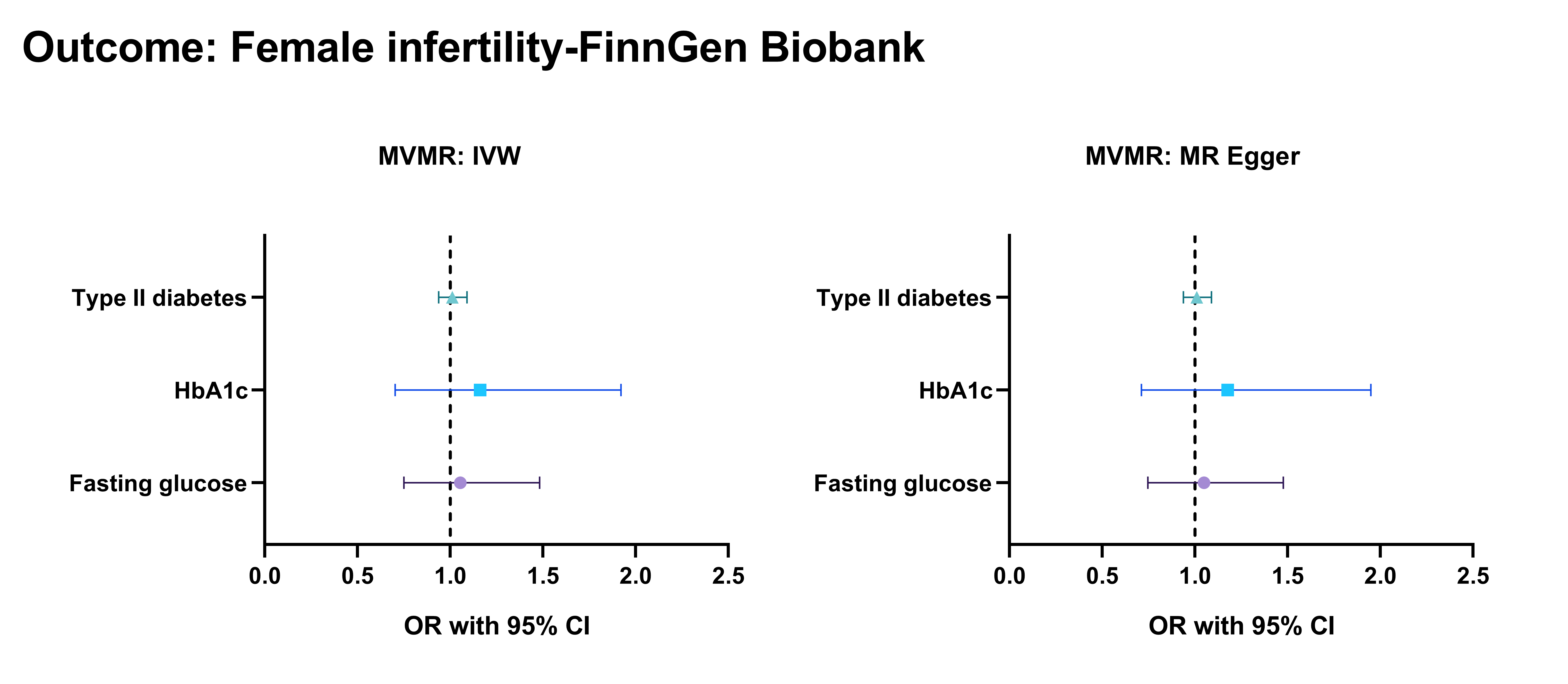


**Figure S7.** MVMR results of the direct effect of three glycemic traits on female infertility (FinnGen Biobank) outcome.

MVMR: multivariate Mendelian randomization; HbA1c: glycated hemoglobin; IVW: inverse variance weighted; MR Egger: Egger's regression for Mendelian randomization; OR: odds ratio; 95% CI: 95% confidence interval.


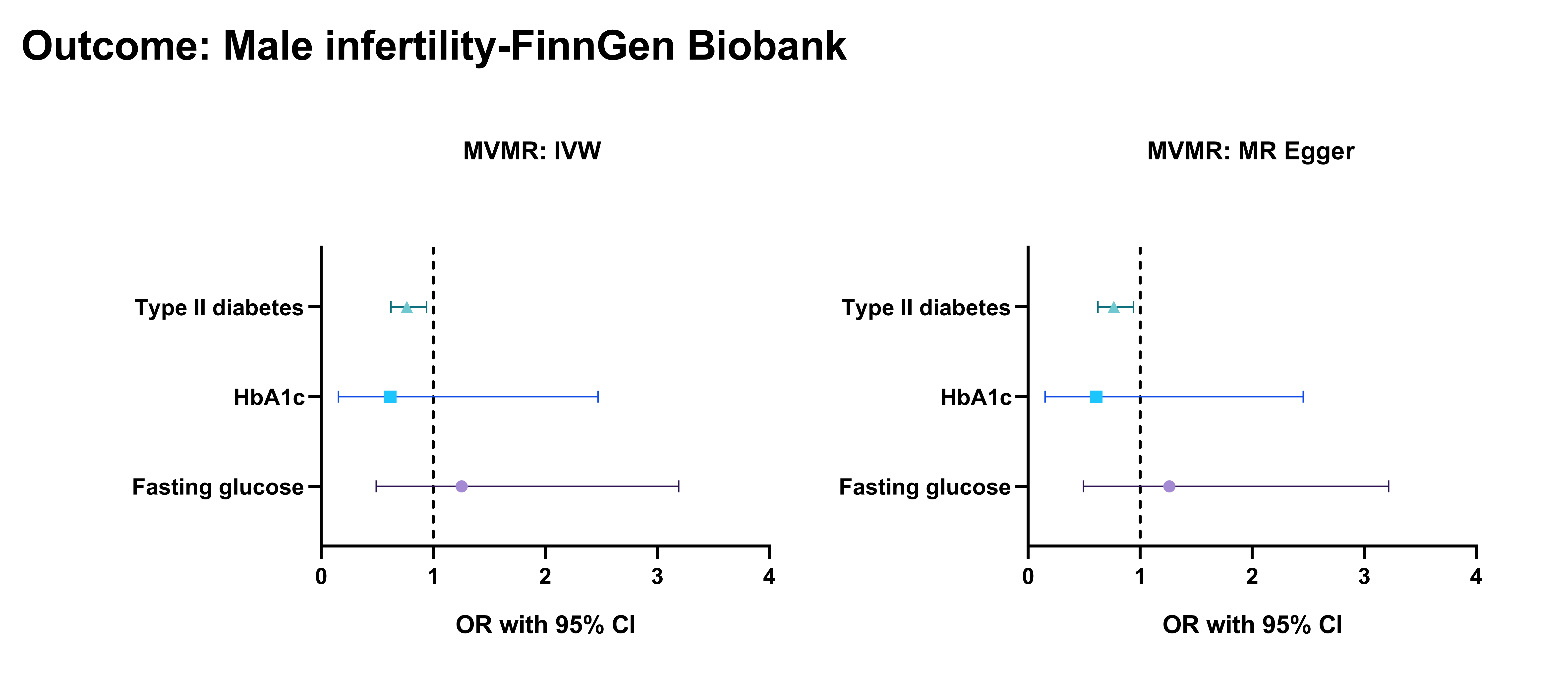


**Figure S8.** MVMR results of the direct effect of three glycemic traits on male infertility (FinnGen Biobank) outcome.

MVMR: multivariate Mendelian randomization; HbA1c: glycated hemoglobin; IVW: inverse variance weighted; MR Egger: Egger's regression for Mendelian randomization; OR: odds ratio; 95% CI: 95% confidence interval.


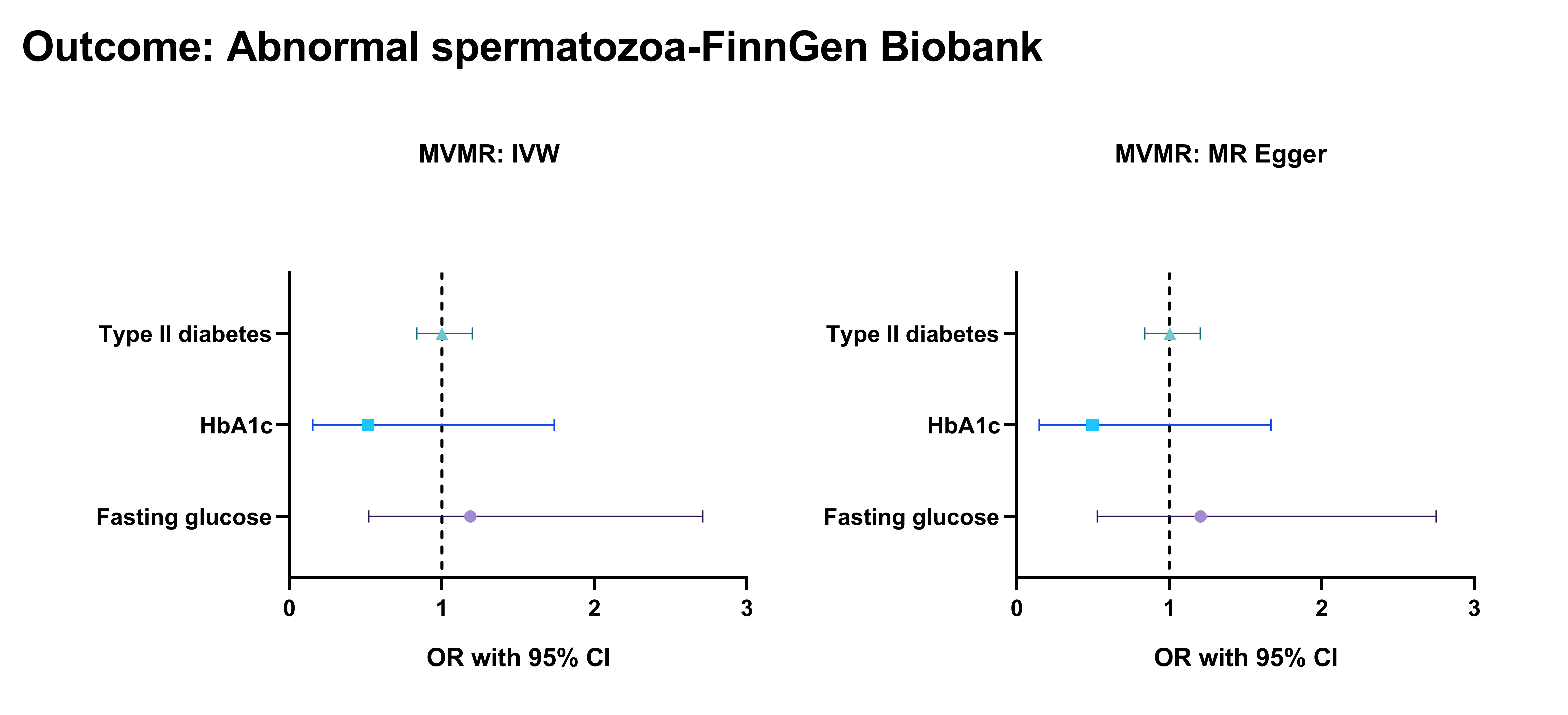


**Figure S9.** MVMR results of the direct effect of three glycemic traits on abnormal spermatozoa (FinnGen Biobank) outcome.

MVMR: multivariate Mendelian randomization; HbA1c: glycated hemoglobin; IVW: inverse variance weighted; MR Egger: Egger's regression for Mendelian randomization; OR: odds ratio; 95% CI: 95% confidence interval.


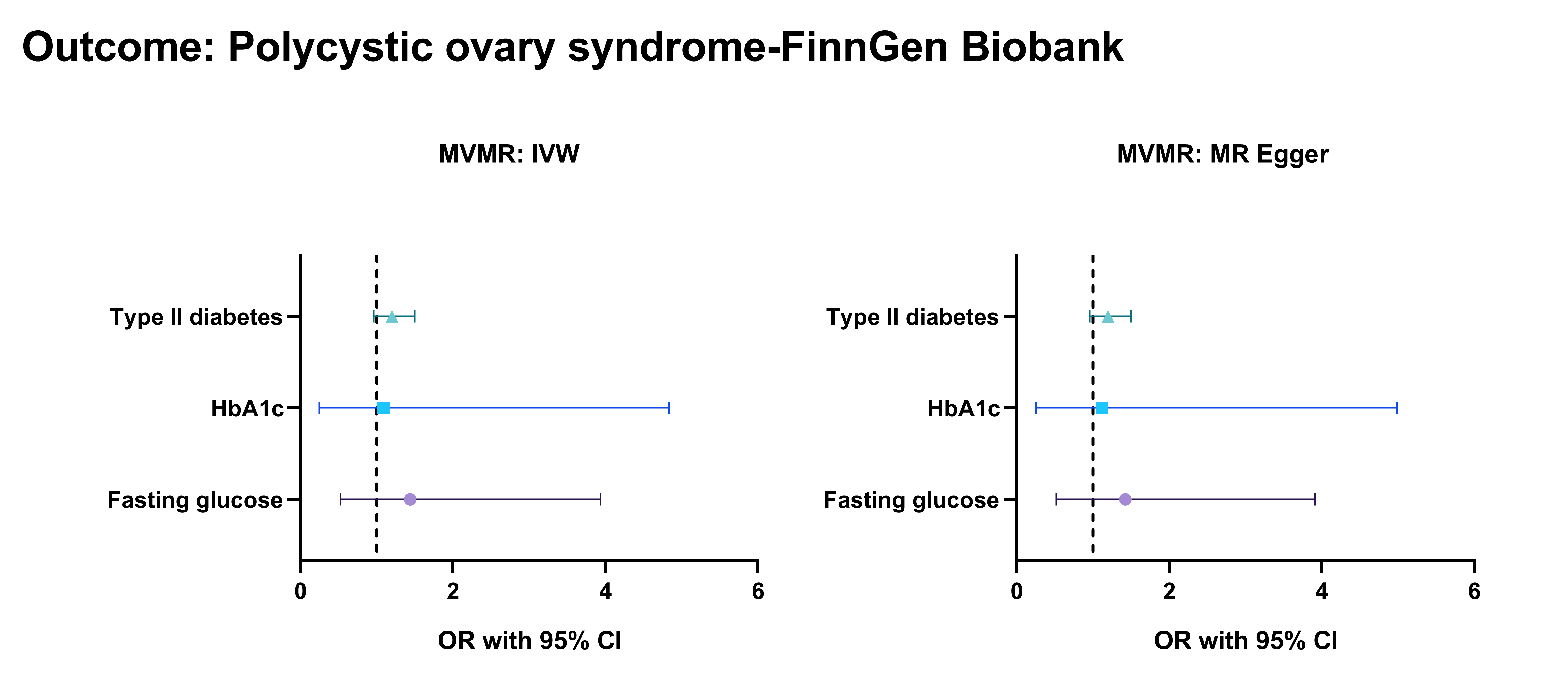


**Figure S10.** MVMR results of the direct effect of three glycemic traits on polycystic ovary syndrome (FinnGen Biobank) outcome.

MVMR: multivariate Mendelian randomization; HbA1c: glycated hemoglobin; IVW: inverse variance weighted; MR Egger: Egger's regression for Mendelian randomization; OR: odds ratio; 95% CI: 95% confidence interval.
